# Differential and coherent processing patterns from small RNAs

**DOI:** 10.1038/srep12062

**Published:** 2015-07-13

**Authors:** Sachin Pundhir, Jan Gorodkin

**Affiliations:** 1 Center for non-coding RNA in Technology and Health, IKVH, University of Copenhagen, Grønnegårdsvej 3, 1870, Frederiksberg C, Denmark

## Abstract

Post-transcriptional processing events related to short RNAs are often reflected in their read profile patterns emerging from high-throughput sequencing data. MicroRNA arm switching across different tissues is a well-known example of what we define as differential processing. Here, short RNAs from the nine cell lines of the ENCODE project, irrespective of their annotation status, were analyzed for genomic loci representing differential or coherent processing. We observed differential processing predominantly in RNAs annotated as miRNA, snoRNA or tRNA. Four out of five known cases of differentially processed miRNAs that were in the input dataset were recovered and several novel cases were discovered. In contrast to differential processing, coherent processing is observed widespread in both annotated and unannotated regions. While the annotated loci predominantly consist of ~24nt short RNAs, the unannotated loci comparatively consist of ~17nt short RNAs. Furthermore, these ~17nt short RNAs are significantly enriched for overlap to transcription start sites and DNase I hypersensitive sites (p-value < 0.01) that are characteristic features of transcription initiation RNAs. We discuss how the computational pipeline developed in this study has the potential to be applied to other forms of RNA-seq data for further transcriptome-wide studies of differential and coherent processing.

Post-transcriptional processing (RNA processing) is a mechanism by which the primary transcripts are processed to produce functional RNA fragments. Alternative splicing and biogenesis of non-coding RNA have been suggested as two important components of RNA processing^1^, which in turn contributes to the diversity of transcriptome inside a cell. These are essential components that act as a significant force in the evolution of phenotypic complexity in mammals^2^,^3^ and have been subject to intense study in recent years^4^,^5^,^6^,^7^. It has already been established from the studies of alternative splicing that the isoform of a transcript (mRNAs as well as some long non-coding RNAs, lncRNAs) adds to the regulatory complexity beyond differential expression, for example by tissue specific alternative splicing^8^,^9^. Notably, these isoforms can sometimes be characterized by a completely non-overlapping sequence, as it is the case for the *PKM* gene, whose alternatively spliced isoforms are comprised of two non-overlapping exons of the gene^10^.

Similar to alternative splicing, biogenesis of non-coding RNAs (ncRNAs) can also be fine-tuned through alteration in the post-transcriptional processing mechanism. We here refer to this phenomenon as *differential processing*. A good example of such an alteration is observed during the process termed as ‘arm switching’ in which the premiRNA switches the arm from where the mature miRNA is processed^11^,^12^. However, a key question is whether differential processing is found in other types of transcripts and to which extent. Here, we address this question within the context of small RNAs. Furthermore, given that many transcripts can also have only a single isoform and can be coherently processed with well defined start and end positions, for example the premiRNAs having a consistent dominant arm^13^, we also address how widespread such *coherently processed* transcripts exist within the context of small RNAs.

Notably, much of the regulation in the biogenesis of non-coding RNAs (ncRNAs) has been studied by comparing their expression profiles between multiple tissue samples^14^,^15^ and has mostly been focused on miRNAs, even though emerging studies involve long ncRNAs (lncRNAs)^16^,^17^. Since, differential processing can potentially be independent of the expression level, it is important to analyze this phenomenon using the actual patterns that originate when short RNAs (reads) generated during ncRNA processing are sequenced and mapped back to the host genome. These patterns, referred to as ‘*read profile*’ or ‘*block group*’, represent the positional arrangement of reads mapped at a locus and are characterized by distinct clusters of reads with similar start and/or stop position referred to as block^18^ (Supplementary Fig. S1). They are often influenced by chemical modifications like in case of tRNAs^19^ or by secondary structure like in case of miRNAs. The read profiles have been utilized to distinguish between major ncRNAs such as miRNA, snoRNA and tRNA^18^,^20^. Tools such as miRDeep2^21^ and miRDeep* 22 employ the read profiles along with the secondary structure potential to predict miRNAs. Likewise, the primary online repository of miRNAs, miRBase has also exploited the read profiles to provide detailed evidence of miRNA annotations^23^. In this study, we extract read profiles from short RNA-seq experiments performed on nine human cell lines and analyze them for differential and coherent processing using a novel computational approach. We determine distinct characteristic features such as read length, ncRNA annotation and regulatory elements, such as transcription start sites associated with differential and coherently processed read profiles. We also show that the read profiles are reproducible between experiments performed on the same tissue in the same as well as different laboratories. Even though we employ our approach on small RNA-seq data, we discuss its potential on other forms of RNA-seq data by showing two examples of differential processing that we observed upon the analysis of total RNA-seq data.

## Results

### Differential processing analysis

We analyzed the block groups or read profiles derived from the ENCODE dataset^24^,^25^, which is comprised of short reads sequenced from the two biological replicates of nine human cell lines (Supplementary Table S1). The analysis of the read profiles from the two biological replicates was performed separately (independent) as well as together (combined) using a pipeline developed for differential processing analysis (see methods). During the combined analysis, we analyzed 701 loci resulting from the conservative requirement that a block group or read profile must be observed in both the replicates of all nine cell lines (see Methods for details). Similarly, we analyzed 778 and 911 loci from replicate 1 and 2 independently, again with the requirement that a block group is observed in all the nine cell lines of the respective replicates. Our aim is to determine the extent by which the read profiles at 701 loci are distinct for a subset of cell lines from the rest (differentially processed loci; DPL) or are completely similar across all cell lines (coherently processed loci; CPL). We used the DPL and CPL identified from the independent analysis of 778 and 911 loci to measure the agreement between the predictions from those identified from combined analysis of 701 loci.

We predict a genomic locus as differentially processed, if the read profiles for a subset of cell lines are dissimilar in comparison to those from rest of the cell lines. The dissimilarity is measured in terms of a cluster score that measures how well separated the read profiles are within a cluster to read profiles outside the cluster (see methods for details). We took an empirical threshold of 0.15 for cluster score, which was determined after comparing the frequency distribution of cluster scores obtained after the differential processing analysis of read profiles from the same cell lines and different cell lines, respectively (see Supplementary Fig. S2). We observed only 1% differentially processed loci from the analysis of read profiles between the same cell line in comparison to 14% such loci identified from the analysis between different cell lines (Supplementary Methods), suggesting that the read profiles between the same cell line are seldom so different that they can lead to a cluster score above the chosen threshold of 0.15. High reproducibility between RNA-seq experiments from same as well as different laboratories has also been observed previously^26^,^27^.

### Differential processing is mostly observed at loci annotated as miRNA, snoRNA and tRNA

**Figure 1:**
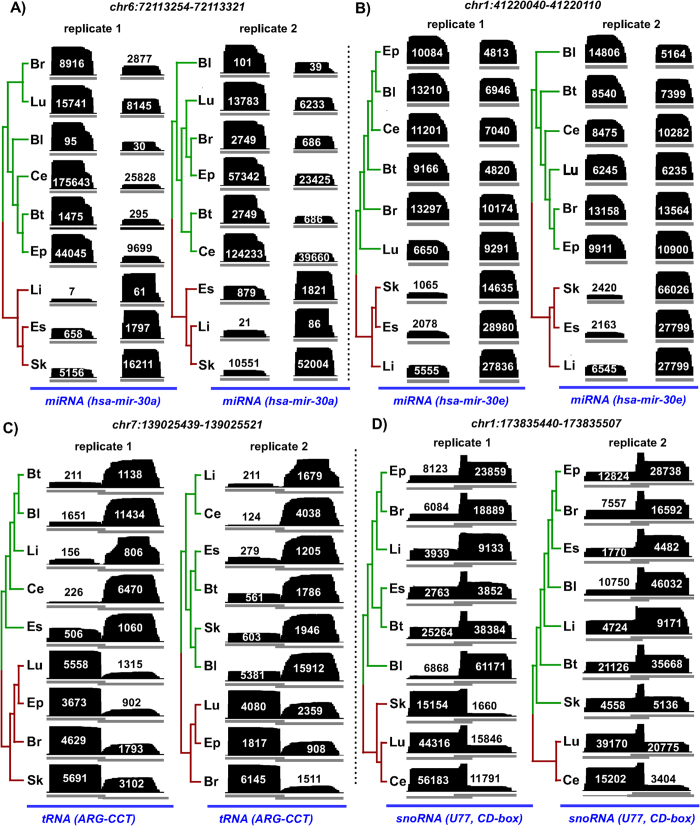
Examples of differentially processed loci (DPL) obtained after the analysis of short total RNA-seq data from nine human cell lines (two biological replicates each). Each panel shows the read profiles (in black) across nine cell lines (both replicates), along with the normalized expression of constituent read blocks (Equation 4). Tree branches corresponding to the distinct clusters of cell lines (p-value < 0.05) are marked with different colors. In all the four examples, we observe two distinct set of read profiles characterized by high expression from one end of the read profile in a sub-set of cell lines and from another end in rest of the cell lines. (**A**,**B**) MiRNA read profiles exhibits a typical example of a process called ‘arm switching’ where pre-miRNA switches the arm from where the mature miRNA is processed^11^. Similar read profiles are observed in both the biological replicates despite much variability in their expression. (**C,D**) Transposition in the expression from the two ends is also observed for tRNA and snoRNA loci. These and rRNAs have also been shown to produce small 5′ and 3′ end fragments in an asymmetric manner that predominantly favors either the 5′ or 3′ end^34^. In snoRNA profile, due to high variation between the read profiles from the two biological replicates of skin, we observed inconsistent cluster scores of 0.19 and 0.14 in replicate 1 and 2, respectively. The nine cell lines from ENCODE are abbreviated with the initials of the parent human tissues (Bl: blood, GM12878; Br: brain, SK-N-SH RA; Bt: breast, MCF-7; Ce: cervix, HeLa-S3; Ep: epithelium, A549; Es: embryonic stem cell, H1-hESC; Li: liver, HEPG2; Lu: lung, AG04450; Sk: skin, BJ).

Out of 701 loci analyzed, differential processing was observed for 97 loci (DPL). Although about one-third of the 701 loci were unannotated (216 out of 701, 31%), only a marginal proportion (three out of 216, 1%) of them were differentially processed (Supplementary Table S2). In contrast, almost all DPL (94 out of 97) were annotated to non-coding RNAs (22 miRNAs, 31 snoRNAs, 30 tRNAs and 11 other ncRNAs). Figure 1 illustrates four examples of DPL observed in the ENCODE dataset. The first two examples are miRNAs namely, hsa-mir-30a and hsa-mir-30e, which have a distinct expression profile between the two arms of pre-miRNA (5′ and 3′ end) across cell lines. While the 5′ end is more expressed in the majority of cell lines, the expression at 3′ end is more pronounced in HEPG2 (liver), H1-hESC (embryonic stem cell) and BJ (skin) for hsa-mir-30a. This is a known example of ‘arm switching’, where pre-miRNA switches the arm from where the mature miRNA is processed^11^,^28^. Similar arm switching is also observed for hsa-mir-30e, which also has previously been reported using PCR-based method for miRNA quantification^28^. Intriguingly, both hsa-mir-30a and hsa-mir-30e, which belong to the same miRNA family (mir-30), are differentially processed between similar set of cell lines (HEPG2, H1-hESC and BJ) and have both been shown to be implicated in epithelial-mesenchymal state transition (EMT) in human pancreatic cells^29^. In total, we identified 22 differentially processed miRNA loci, of which nine are clear cases of ‘arm switching’ (Supplementary Table S3) and the remaining 13 cases exhibit ‘arm loss’. Note that all these cases fulfill the requirement of expression in both replicates of all nine cell lines. Five out of the nine clear cases of ‘arm switching’ have previously been reported, three in human and two in mouse (Supplementary Table S3). From the opposite perspective, a total of 39 cases of ‘arm-switching’ have been reported in human, of which five fulfilled the expression criteria and are thus in our input set. Four of them are recovered, three as arm switched and one as arm loss (Supplementary Table S4). Also, none of the remaining 12 cases of arm-loss correspond to 33 previously known cases of arm switching in mouse^30^,^31^.

In addition to miRNAs, we also observed differential processing in 31 snoRNAs, 30 tRNAs and 11 other ncRNAs (Supplementary Table S2). The third and fourth example in Fig. 1 illustrates differential processing (DP) at loci encoding for a tRNA and snoRNA, respectively. Similar to miRNAs, both loci are characterized by transposition in expression from the two ends of its read profile (Fig. 1C). Preferential processing of small RNAs (19 nt) from the 5′ end of tRNAs was first reported in Arabidopsis^32^ which was later supported by another genome-wide study^33^. Also in human and mouse, tRNAs, snoRNAs, rRNAs and snRNAs have been shown to produce 5′ and 3′ end short RNA fragments in an asymmetric manner that predominantly favors either the 5′ or 3′ end^34^,^35^. Many of these fragments, specifically those processed from tRNAs, have been shown to be capable of down regulating target genes *in vitro*^34^. Inherent consistency of read profiles, composed of small RNA fragments between the two biological replicates further suggests that, besides miRNAs, structural ncRNAs such as snoRNAs, tRNAs and rRNAs can also be processed to produce shorter RNA fragments. The graphical illustration of the read profiles corresponding to all of the 97 DPL predicted on the combined analysis of the two biological replicates is available in the supplementary file.

### miRNA arm switching and its effect on the expression of target genes

**Figure 2:**
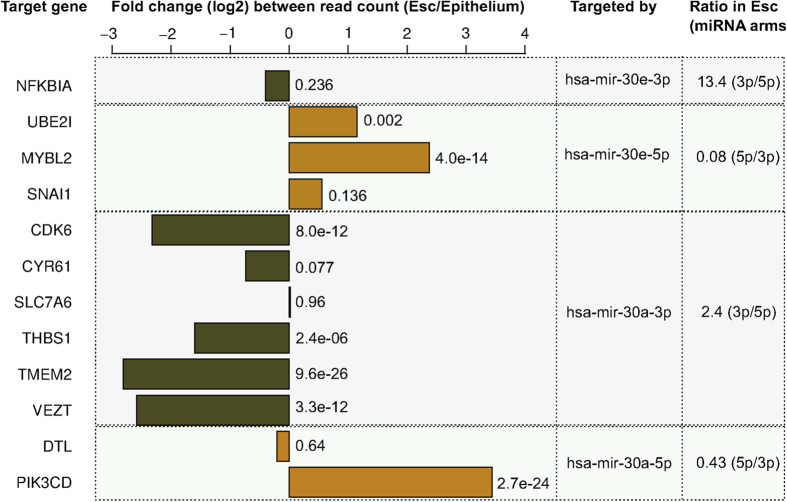
The fold change (log2) in the expression of twelve genes targeted by either of the two differentially processed miRNAs, has-mir-30a and has-mir-30e. In Embryonic stem cell (Esc), the 3p-arm of both the miRNAs is dominantly expressed. Consequently, we see a decrease in the expression of genes targeted by 3p-arm (green bars) in Esc. In contrast, the genes targeted by the 5p-arm, which is dominantly expressed in epithelial cells, show increase in their expression (yellow bar) in the Esc. A significant difference in the expression is computed using DESeq2^37^ and the significance level (p-value) is indicated adjacent to each bar.

We further analyzed the expression of twelve genes, which are targeted by either of the two differentially processed miRNAs (hsa-mir-30a and hsa-mir-30e; Fig. 1A,B). The miRTarBase database that contains the experimentally validated miRNA-target interactions was used to determine the twelve target genes^36^. These twelve genes were selected because they have been validated by three independent experimental methods (luciferase reporter assay, qRT-PCR and western blot)^36^. The fold change in the expression of the target genes was compared, using DESeq2^37^ between embryonic stem cell (H1-hESC) and epithelial cell (A549) lines in which the alternative arms of both the miRNAs are dominantly expressed (Fig. 1A,B). To determine the expression of the genes in H1-hESC and A549 cell lines, we used the corresponding polyA long RNA-seq read data sets, downloaded from the ENCODE project^24^ in SAM format^38^. For all the genes, except *DTL*, we observed an anti-correlation between the expression of miRNA arm (5p or 3p) and the target genes (Fig. 2), which is a characteristic feature of miRNA-target interaction. Interestingly, one of the key genes, *SNAI1* involved in epithelial-mesenchymal transition and target of the miRNA, hsa-mir30e-5p is also highly expressed in H1-hESC in comparison to A549. Although the higher expression of *SNAI1* in H1-hESC does not reach the significance level of <0.05, an apparent increase in its expression relative to that in the A549 cell line is in agreement with the role of *SNAI1* to induce epithelial to mesenchymal transition by repressing the expression of E-cadherin protein. E-cadherin is highly expressed in epithelial cells and is responsible for maintaining cell adhesion, a primary feature of epithelial cells. Thus, the lower expression of hsa-mir-30e-5p and the up-regulation of target gene, *SNAI1* in H1-hESC as compared to in the A549 cell line supports the role of hsa-mir-30e in epithelial-mesenchymal transition as suggested in an earlier study^29^.

### Read profiles are reproducible and robust against local sequence context and expression variation between samples

To measure the extent of reproducibility between read profiles, we compared them between pair of short RNA-seq experiments performed on the same tissue as well as between different tissues, respectively. The analysis was replicated for experiments performed in same as well as different laboratories (see Supplementary results and Supplementary Table S5 for details). We observed a significantly higher percentage (95% at p-value <0.01, Fisher’s exact test) of short RNAs, which exhibit reproducible read profiles between short RNA-seq experiments performed on the same tissue in comparison to those performed between different tissues (Supplementary Fig. S3, S4 and Results). Thus, our analysis agrees with the idea that a read profile of a transcript is a reproducible phenomenon and by being more consistent between replicates of the same tissue, it often represents the processing mechanism of the host transcript.

Owing to the reproducibility of read profiles, we observed a high correlation (*R*^*2*^ of 0.73 to 0.8) between the cluster scores obtained after the analysis of the two replicates of the ENCODE dataset independently and after combining them together (Supplementary Fig. S5, S6 and Results). Most DPL were having consistently high cluster scores between the two sets of analysis (independent and combined) suggesting robustness of the proposed method (Supplementary Fig. S5, Supplementary Table S6 and Results). We also analyzed the dependency of cluster score and deepBlockAlign alignment score on the number of blocks within the reads profiles. We observed a negative correlation (*R*^2^ = −0.61; p-value = 4.0e-37) between the alignment score and number of blocks, presumably due to higher entropy in the positional arrangement of reads^18^ within read profiles containing higher number of blocks. Indeed, a positive correlation (*R*^2^ = 0.43; p-value = 4.2e-13) was observed between the entropy and number of blocks. However, we did not observed any correlation between the cluster scores and number of read blocks (*R*^2^ = −0.05; p-value = 0.38), suggesting it to be robust towards the number of blocks within the reads profiles at a locus.

The GC content and various dinucleotide frequencies related to GC content have been shown to bias the transcript coverage^39^,^40^. Upon investigation, we observed no significant difference (Kolmogorov-Smirnov test, p-value <0.05) in the density distribution of the nucleotides between differentially processed and background loci (Supplementary Fig. S7–S11 and Results). Also, the analysis after accommodating for bias effect gave similar results (Supplementary Fig. S12). Similarly, the alternatively processed forms of transcripts from 97 DPL were not influenced by their expression difference between samples (Supplementary Results).

### Coherent processing analysis

**Figure 3:**
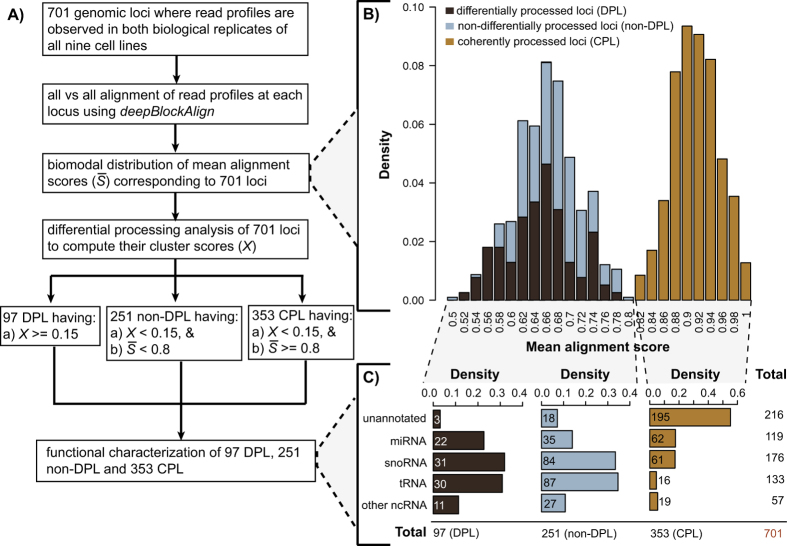
Analysis of read profiles from 701 genomic loci (*L*), their mean alignment scores (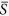) and its relationship to non-coding RNA processing and annotation. (**A**) General outline of various analysis steps performed to identify differentially and coherently processed loci. (**B**) Mean alignment score is computed by all versus all alignment of read profiles at each of the 701 loci using deepBlockAlign and computing their mean. A bimodal distribution is observed where all 97 differentially processed loci (DPL) and 251 non-DPL have a mean alignment score of <0.8, while the rest 353 loci have a mean score of ≥0.8. Here, a high mean alignment score (≥0.8) indicates coherent read profiles across all cell lines at a locus (coherently processed loci; CPL). Non-DPL refers to those loci where the read profiles are neither differentially nor coherently processed. (**B**) Density distribution of the non-coding RNA annotations belonging to each of the three categories of genomic loci (DPL, non-DPL and CPL). Coherently processing (CPL) is enriched in unannotated loci (p-value = 5.8e-07, Fisher’s exact test). In contract, most (83 out of 97) DPL are annotated (see methods) for the three ncRNA classes (miRNA, snoRNA and tRNA).

For each of the 701 loci where block groups are observed in both replicates of nine cell lines from the ENCODE dataset, we aligned all block groups against each other using deepBlockAlign^18^ and computed the mean alignment score (Fig. 3A). We observed a bimodal distribution characterized by around half of the loci (353) having a mean alignment score of ≥0.8 and the rest of the 348 loci having a mean alignment score of <0.8 (Fig. 3B). Since, the alignment score is a measure of similarity between two read profiles, a high mean alignment score (≥0.8) for the 353 loci indicates that the corresponding read profiles are coherent in terms of their respective arrangement of reads across different cell lines. We therefore have termed the 353 loci (alignment score ≥0.8) as Coherently Processed Loci (CPL). To further characterize the distinct features of read profiles or block groups from CPL, we compared the 6,354 block groups from 353 CPL with 6,264 block groups from the rest of the 348 loci (18 block groups per locus due to two replicates for each of the nine cell lines) in terms of the number of blocks, entropy and length of the block groups. Here, entropy measures the randomness in the arrangement of reads within a block group^18^. The lower the entropy, the more precisely arranged are the reads with respect to their start positions within the block group. We observed a significant enrichment (p-value = 5.8e-243, Fisher’s exact test) of 5,371 (85%) block groups that are short and composed of precisely arranged constituent reads at the 353 CPL in comparison to 1,875 (30%) of such block groups at the rest of the 348 loci (Supplementary Table S7). We define a block group as short and precise, if it is composed of only one read block, have low entropy of ≤2 and is ≤40 nt in length.

Interestingly, in contrast to DPL that were mostly annotated, more than half of the CPL (195 out of 353) were unannotated and the remaining 158 CPL were annotated as miRNA, snoRNA, tRNA or other ncRNA (rRNA, snRNA and scRNA) as shown in Fig. 3C. Furthermore, almost all (346 out of 353) CPL were observed within non-exonic regions of the genome (116 in intron, 67 in 5′ UTR, 49 in 3′ UTR and 114 in intergene) and were significantly enriched in 5′ UTR (p-value = 0; Binomial test) and 3′ UTR regions (p-value = 5.03e-97; Binomial test) (Supplementary Methods).

### Annotated CPL encode for short RNAs that are preferentially biased in terms of their position towards the 5′ or 3′ end of the corresponding non-coding RNA

Among the 158 annotated CPL, 62 miRNA CPL, as expected, were composed of a single read block corresponding to the mature arm and were missing the reads corresponding to the passenger arm mainly due to low expression (<10%) relative to total expression of the miRNA block group. However, we did not observe any preference in the position of the mature arm with respect to location in either the 5′ or 3′ end of the pre-miRNA (Supplementary Fig. S13A). Similar to miRNAs, almost all of the other ncRNAs associated with CPL were composed of a single block of reads, albeit different in terms of the specific preference in the position of the read block within the ncRNA gene (Supplementary Fig. S13B–E). More specifically, the read block within the 61 CPL snoRNAs (48 C/D-box snoRNAs and 13 H/ACA-box snoRNAs) was located exclusively at the 5′ end (Supplementary Fig. S13B,C). For 16 tRNAs, the read block was observed at both the 5′ and 3′ end, however most were preferentially located at the 5′ end (Supplementary Fig. S13D). The read block was located preferentially towards the 3′ end of the 13 rRNAs (Supplementary Fig. S13E).

The generation of small RNA fragments (>18 nt)^34^,^41^,^42^,^43^,^44^,^45^ and their preferential bias towards the 5′ or 3′ end of these ncRNA classes have also been observed in recent studies^32^,^34^,^35^. In agreement with the observation by Li *et al.*^34^, we also observed that a major fraction of reads within the tRNA genes is generated from the 5′ end. These small RNA fragments are largely independent of the canonical RNA interference (RNAi) processing machinery and are involved in diverse biological processes^34^,^46^. Although it is tempting to assume the small reads processed from tRNAs and rRNAs as degradation products, mainly due to the high expression of these ncRNAs within the cell, a recent study has shown that tRNAs and rRNAs undergo stress induced cleavage to produce stable small RNAs, and this mechanism is conserved from yeast to human cells^47^. Furthermore, the small reads from tRNAs have also been shown to associate with Ago2 and down regulate the target genes by transcript cleavage *in vitro*^34^. Similarly, the small RNAs processed from snoRNAs have been shown to have functional role in the regulation of either splicing, or translation^35^,^42^,^48^. The precise processing (low entropy of the read block) and complete consistency (high deepBlockAlign score) in the read profiles composed of these small reads across multiple human cell lines further support the functional relevance of these small RNAs. The graphical illustration of the read profiles corresponding to all of the 153 annotated CPL predicted on the combined analysis of the two biological replicates is available in the supplementary file.

### Unannotated CPL encode for short RNAs that are enriched for co-location to Transcription Start Sites (TSS) and transcribed regions in the human genome

Out of 216 unannotated loci analyzed, 195 (90%) loci were observed to harbor coherent read profiles (p-value = 5.8e-07, Fisher’s exact test, Fig. 3C). We have referred to a locus as unannotated, if it is devoid of any non-coding RNA annotation (see Methods). To determine the functional annotation corresponding to 195 unannotated CPL, we performed an enrichment analysis of CPL against the ENCODE genome segmentation tracks in which the human genome has been divided into seven distinct chromatin states representing regulatory and functional activity of genomic regions^49^,^50^ (Supplementary Methods). The enrichment analysis was performed using the simple binomial model where we compare the frequency by which the CPL overlap to a chromatin state with the frequency that is expected under the null model of throwing a dart onto the genome^51^ (Supplementary Methods).

**Figure 4:**
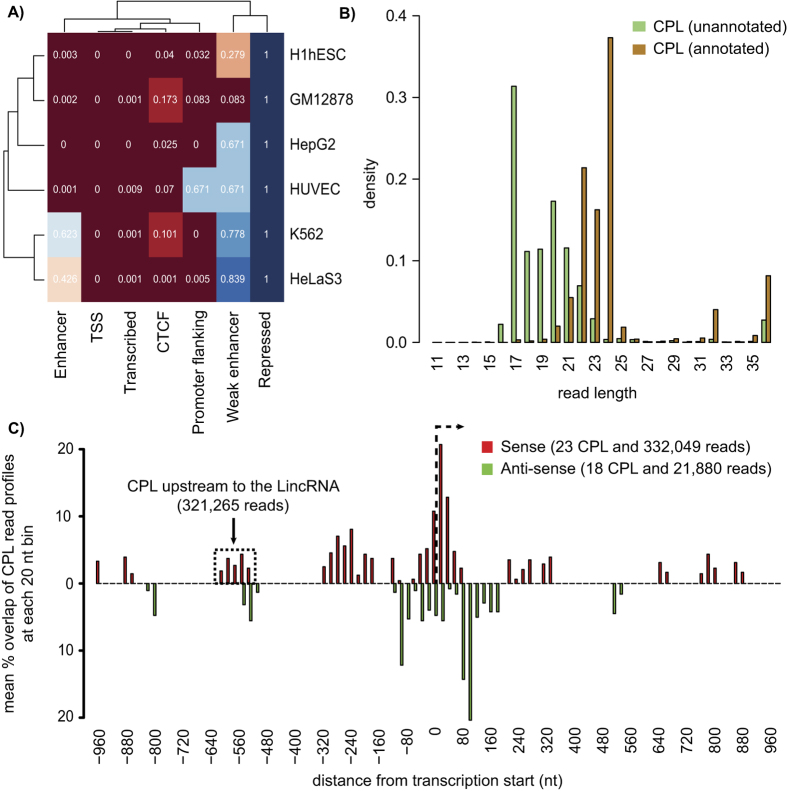
Read length and genomic location of read profiles from 195 unannotated and coherently processed loci (CPL) in the ENCODE dataset. (**A**) The 195 CPL are consistently enriched (binomial test, p-value < 0.01; number in the boxes) for co-location to transcription start sites (TSS) and transcribed regions (x-axis) across all six human cell lines (y-axis). In all the six cell lines, the whole human genome has been divided into seven genomic distinct regions (enhancer, TSS, transcribed, CTCF, promoter flanking, weak enhancer and repressed) using ChIP-seq data from the ENCODE project^49^,^50^. (**B**) Two distinct distributions of read length from 195 unannotated and 158 annotated CPL, respectively were observed. While most reads from annotated CPL were ≥22 nt in length, unannotated CPL were observed to be comprised mostly of reads that are <22 nt in length. A CPL is termed as annotated, if its genomic coordinates overlaps to the coordinates of a ncRNA and unannotated otherwise. **C**) Out of 195 unannotated CPL, read profiles from 41 loci are observed to be in proximity (1000 nt up- or downstream) to TSS and the reads from these loci are mostly observed to be either upstream or overlapping (−650 to 120 nt) to the TSS. Displayed is the mean percentage overlap of 41 read profiles (y-axis) at each of the 20 nt bin into which the genomic region 1000 nt up- and down-stream of TSS has been divided (x-axis). The bars above and below the x-axis show the coverage of those read profiles, which are in sense and anti-sense direction to that of the transcription, respectively. The black vertical line indicates the transcription start site and black arrow depict the direction of transcription.

Out of the seven distinct chromatin states, we observe a significant enrichment of the CPL within two states, TSS and transcribed region in all of the six cell lines from which they were generated (p-value < 0.01, Binomial test, Fig. 4A). In total, 107 out of 195 CPL were observed to be overlapping to a TSS (23) or a transcribed region (84) in at least one out of the six cell lines. Recently, a novel class of non-canonical miRNAs derived from transcription start site of protein coding genes (TSS-miRNAs) has been discovered in mouse^52^. However, we did not observed an enrichment of 195 CPL with the orthologous TSS-miRNAs in the human genome (p-value = 0.28, Binomial test).

**Figure 5:**
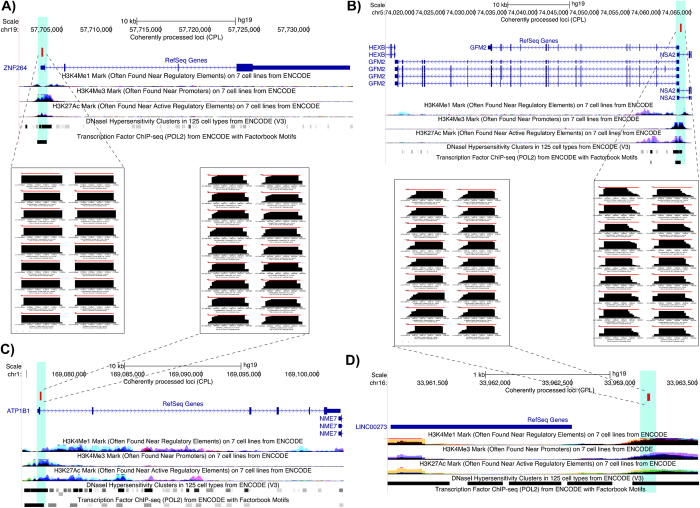
Representative examples of coherently processed loci (CPL). UCSC genome browser view showing the genomic location of four CPL (**A–D**). All the CPL (marked in red) are located at active promoter or enhancer regions (highlighted in green) as supported by an enrichment of H3K4me3 or H3K4me1 histone modification (signal height), enrichment of DNAase I hypersensitivity clusters (black bars) and presence of POL2 binding site (black bars). Also shown are the read profiles corresponding to four CPL (inset) organized in nine rows (cell lines) and two columns (replicates). As evident the read profiles are similar across both replicates of nine cell lines.

Next, we analyzed the enrichment of 195 CPL for short and long ncRNAs generated due to the bidirectional nature of the transcription initiation at active TSS and enhancer regions^53^,^54^,^55^,^56^,^57^,^58^. These ncRNAs include small (<22 nt) ncRNAs, synonymously termed as transcription initiation RNA (tiRNAs) or transcription start site-associated RNAs (TSSa-RNAs)^53^,^54^,^57^ and long ncRNAs, such as promoter-upstream transcripts (PROMPTs) or long non-coding RNAs (lncRNAs)^57^,^58^ generated from the bidirectional TSS. The tiRNAs or TSSa-RNAs, in particular, are derived from nascent RNAs protected by stalled RNAPII against nucleolysis^57^. To determine the possible association of 195 unannotated CPL with tiRNAs, we compared the length of reads from these CPL with those mapped to 158 annotated CPL and observed two completely distinct distributions. While most of the reads from annotated CPL were >22 nt with a modal length of 24 nt, most reads from unannotated CPL were <22 nt having a modal length of 17 nt (Fig. 4B), thus agreeing with the tiRNAs in terms of the read length. Next, we analyzed the location of the unannotated CPL with respect to the TSS and observed 41 CPL to be located within a window of 1000 nt upstream and downstream to the TSS (Fig. 4C). The TSS are determined using the 5′ end of the gene annotations available from the GENCODE project^59^. Specifically, we divided the 2000 nt sized window around TSS into 100 equally sized bins of 20 nt each and computed the percentage overlap of a CPL at each bin. The percentage overlap of each bin is then averaged over all the 41 CPL. In agreement with Taft *et al.*^54^, we observed CPL in the sense direction peaking at the 50 nt downstream to the TSS. Furthermore, similar to anti-sense tiRNAs reflecting bidirectional promoters, many CPL were also located upstream to the TSS in anti-sense direction. However, unlike tiRNAs, we also observed CPL located up to 650 nt upstream to the TSS in sense direction and 100 nt downstream to the TSS in anti-sense direction (Fig. 4C). Figure 5 shows four representative examples of CPL located at or in proximity to TSS or enhancer regions. The TSS or enhancer regions are supported by an enrichment of H3K4me3 or H3K4me1 histone modification, enrichment of DNAase I hypersensitivity clusters and presence of POL2 binding site.

Only 41 out of 195 unannotated CPL were observed near (1000 nt up- or downstream) the TSS, despite that almost all of them are having a read length similar to that of tiRNAs. Also, only four out of 195 unannotated CPL overlapped with the 2,428 gene promoters (p-value = 0.97, Binomial test) studied for enrichment of PROMPTs^57^. We suspect that this is due to the fact that the 2,428 promoters represent a small subset of all human gene promoters, selected on the basis that they should not overlap any other annotated mRNA^57^. Therefore, we also performed an enrichment analysis of the remaining 154 CPL for the DNase I hypersensitive sites (DHS) that reflect an open chromatin structure, a marker to *cis*-regulatory regions bound by transcription factors in the genome. We used the DHS determined based on the DNase-seq experiments performed on 125 cell lines in the ENCODE project^60^ and observed a significant enrichment of 154 CPL for overlap to the DHS (p-value = 3.5e-11, Binomial test). In total, we found 47 CPL overlapping to DHS. As expected, similar enrichment was also observed for all 195 CPL at the DHS (p-value = 8.2e-29, Binomial test). Thus, out of 195 CPL, we observed 88 CPL overlapping DHS (47) or in close proximity to known TSS (41). The graphical illustration of the read profiles corresponding to all of the 195 unannotated CPL predicted on the combined analysis of the two biological replicates is available in the supplementary file.

## Discussion

We use read profiles obtained by analyzing short RNA-seq data from nine human cell lines (two biological replicates each; Supplementary Fig. S1) to determine the prevalence of differential and coherent processing in the human transcriptome (<200 nt). We define a genomic locus as differentially processed (DPL) if the read profiles, which represent the positional arrangement of reads mapped at a locus, are distinct for a subset of cell lines from the rest. On the other hand, a locus is termed as coherently processed (CPL), if the read profiles are similar across all cell lines. We show distinct distributions of differential and coherently processed loci in annotated and unannotated regions of the genome, respectively. While almost all of the DPL are observed in genomic regions annotated for various ncRNAs, especially miRNA, snoRNA and tRNA, CPL are almost equally distributed in annotated and unannotated regions. This suggests that much of the regulation in the processing mechanism of small RNAs happens for annotated ncRNAs that are known to play an important role in the active gene regulation such as miRNA, snoRNA and tRNA^7^,^34^,^61^.

A total of nine clear cases of ‘arm switching’ are observed in miRNA. Two of the observed miRNA ‘arm switching’ cases are from the hsa-mir-30 family (hsa-mir-30a-5p and hsa-mir-30e-5p). These miRNAs are co-transcribed from the intron of genes encoding for transcription factors and have been suggested to be involved in epithelial-mesenchymal transition (EMT) in human pancreatic cells^29^. Specifically, hsa-mir-30e is co-transcribed from the intron of *NF-YC* transcription factor gene and, both of these up-regulates the expression of E-cadherin protein that maintains the epithelial cell phenotype^62^. While *NF-YC* directly up-regulates the expression of the E-cadherin gene, hsa-mir-30e contributes to maintain the epithelial state by binding to the 3′ UTR region of the *SNAIL1* transcript thereby inhibiting its translation, which otherwise acts as a repressor to the *NF-YC*^63^. Furthermore, the regulatory role of the reads expressed from both the arms of hsa-mir-30e has been established using a PCR-based method for miRNA quantification^28^. Upon analyzing the effect on the expression of twelve genes targeted by the two alternate mature arms (5p and 3p) of hsa-mir-30a and hsa-mir-30e, we observe an anti-correlation between the expression of almost all target genes and the mature arm, which is a characteristic feature of miRNA-target interaction.

Apart from the clear cases of ‘arm switching’ in miRNA, many snoRNAs, tRNAs and rRNAs are also observed to have transposition in the expression of reads from their 5′ and 3′ ends across cell lines. The feature of snoRNA, tRNA and rRNA to produce short reads from their 5′ and 3′ ends in an asymmetrical manner has been reported earlier^34^,^35^. Here, we show that this feature is consistent as well as regulated across different human cell lines. This further supports the conclusion of recent studies that these structural ncRNAs are also processed to form functional short RNA fragments^34^,^41^,^42^,^43^,^44^,^45^,^47^,^64^. Employing more relaxed criteria, such as requiring less presence in fewer cell lines than all nine is expected to result in higher sensitivity. Furthermore, besides the differential processing, a part of the difference observed between read profiles might arise from differential stability of the RNA in different cell lines. Here, differential stability refers to the similar transcriptional level of a mRNA but difference in its stability arising due to various posttranscriptional events including processing. Differential stability often leads to differential expression between cell lines^65^.

Similar to DPL, CPL are also observed in regions annotated for various ncRNAs, especially miRNA and snoRNA. Interestingly, ~50% of the miRNAs analyzed are observed to have coherently processed read profiles, primarily composed of one read block corresponding to the same mature arm across all cell lines. This observation is in agreement with an earlier study that has shown that 50% of the pre-miRNAs exhibit the same dominant strand across multiple tissues^13^. In contrast to miRNA, CPL annotated for snoRNA, tRNA and rRNA show position bias in the arrangement of reads from their 5′ or 3′ ends, as it has also been observed in an earlier study^34^. In contrast to DPL, CPL are significantly enriched in unannotated regions. In fact, ~90% (195 out of 216) of the analyzed unannotated regions are observed to harbor coherently processed read profiles. We showed that the reads processed from these 195 unannotated CPL are significantly shorter (<22 nt) and are enriched for transcription start sites and DNase I hypersensitive sites in the human genome. Many of the reads encoded from these loci showed read length and proximity to transcription start sites (TSS) similar to the recently discovered class of small ncRNA, transcription start site-associated RNA (TSSa-RNA) or transcription initiation RNA (tiRNA)^53^,^54^,^55^,^56^.

Several recent studies have shown read profiles as a distinct feature of various non-coding RNAs representing their processing mechanism^18^,^20^,^66^,^67^. However, their reproducibility within biological replicates from the same and different laboratories has never been established, an essential prerequisite for the analysis required in this study. Consequently, we show that read profiles are indeed more consistent between short RNA-seq experiments replicated over the same in comparison to different tissues, both in the same as well as different laboratory. However, at a few loci, we also observe inconsistency between the two biological replicates due to the high variability of read profiles, especially for snoRNA and tRNA. This could be due to the higher dynamics in the processing of the snoRNAs and tRNAs, which at least for snoRNAs has been reported in Windhager *et al.*^1^ where it was shown that many introns encoding for snoRNAs have poor processing efficiency. Furthermore, in comparison to miRNAs, the read profiles for snoRNAs and tRNAs are less precise in the arrangement of constituent reads with respect to their start positions^18^, which also point to high dynamics in their processing mechanism. Although used on small RNA-seq data, our pipeline can be employed on any type of RNA-seq data as exemplified by Supplementary Fig. S14 and S15. In the first example, the differential processing is observed in the 3′ UTR of the *RABEP1* gene using total RNA-seq data^68^. The example supports the expression of an alternate splice form of transcript in several tissues that is comprised of a region otherwise annotated as intron by RefSeq^69^. Intriguingly, the alternate splice form of the transcript is supported by the latest Ensembl annotation^70^ (Supplementary Fig. S14). The second example illustrates the phenomenon of differential processing at a much larger region (~500 nt) that partially overlaps a pseudo gene as per latest Ensembl annotation^70^ (Supplementary Fig. S15). However, the reasoning behind this DPL is yet to be interpreted.

In conclusion, our results highlight the significance of read profiles to understand the processing mechanism and its fine regulation in various non-coding RNAs. We show the extent of differential and coherently processed loci in human genome, and points to various loci, for which the functional annotation is yet to be described. We anticipate that the processing analysis will enter functional studies of genes in parallel to the differential expression analysis. Future directions include experimental validation of some of the differentially processed loci. Also, the relationship between expression of small RNAs processed from unannotated CPL and their proximal gene body will be investigated to study the potential role of these small RNAs as enhancer RNAs. Another useful validation step to the proposed differential processing pipeline can be to compare the expression of processed small RNAs with that of the parent primary transcript using polyA RNA-seq data.

## Methods

### High-throughput RNA-sequencing input data and mapping to reference genome

To select an appropriate dataset for our analysis, we performed a search for short RNA-seq experiments that are performed on two biological replicates of at least six different human tissues in NCBI Gene Expression Omnibus (GEO) database^71^. We selected human for the analysis because it is one of the highly annotated genomes available. We downloaded the only dataset that fulfilled the search criteria and is comprised of 18 short total RNA-seq experiments in BAM format. The selected dataset comprises of 740 million reads and is part of the ENCODE project^24^,^25^ and was prepared by using short total-RNA from nine different human cell lines reflecting tissues of diverse origin (Supplementary Table S1). Each cell line is having two biological replicates and prior to sequencing, these biological replicates have been grown and isolated independently. The sequencing protocol for the short total-RNA aims to sequence RNAs shorter than 200 nt (both capped and 5′ monophosphate) that have not been separated based on the poly-adenalyation method. A complete account of the sequencing and mapping procedure is described in Fejes-Toth *et al.*^55^. In the following, we will refer to this dataset as the ENCODE dataset (Supplementary Table S1). To determine an empirical cut-off at which to consider a genomic locus as differentially processed we downloaded another dataset. This dataset is comprised of six RNA-seq experiments replicated on ribosomal RNA-free total-RNA from human liver cell lines^72^. The aim is to determine the extent of variation between read profiles from RNA-seq experiments performed on different cell lines and same cell lines, respectively. The dataset (GEO id: GSE25028) is also composed of some experiments performed by independent scientists and has earlier been used to study the reproducibility of RNA-seq experiments^72^. The dataset was downloaded in FASTQ format from GEO^71^ and, hereafter, we will refer to this dataset as the Raz dataset (Supplementary Table S8). Since this came in raw FASTQ format, the Raz dataset was quality filtered (removal of linkers and adapters, minimum read length of 18 nucleotides, trim nucleotides in reads with quality score less than 20) using FASTX-Toolkit^73^. The human (hg19, Feb. 2009) genome assembly, obtained from the UCSC genome browser^74^, served as the respective reference for read mapping using segemehl^75^ with default parameters. The segemehl software detects mismatches and indels and reports multiple hits with optimal score. To account for sequencing errors and ncRNA editing effects^19^, we required a minimum mapping accuracy of 85%.

### Processing of mapped reads to define block groups or read profiles

Post read mapping, we identified the distinct accumulation of uniquely mapped reads for each cell line by assigning two reads to the same locus when they are separated by less than 50 nt. Then, to detect specific read profiles, we divided consecutive reads within these loci into blocks using blockbuster (with parameters: -distance 50, -minBlockHeight 10, -minClusterHeight 10, -scale 0.5 -blockHeight rel)^76^. blockbuster merges mapped reads into blocks based on their location in the reference genome (Supplementary Fig. S1). Thus, stacks of reads are combined to read blocks, which is analogous to tags (set of reads) processed from a specific locus. We chose 50 nt as the threshold to consider two reads from separate loci because the mean length of reads in our two datasets is 40 nt. Therefore, two genomic loci separated by a region of >50 nt with no mapped reads can most likely be considered as distinct. The obtained sets of one or more read blocks at a locus are then called block groups or read profiles (Supplementary Table S1 and S8). We used a relative blockbuster score cut-off of 10% rather than an absolute cut-off. This means that only those blocks that have at least 10% reads relative to the total number of reads in a block group are included within the block group. This is done in order to make the read profiles from different cell lines that may have been sequenced to variable sequencing depths, comparable. Here after, we will use the term block group and read profile synonymously.

All the block groups were then compared to known annotations (1049 microRNA loci from miRBase v16^23^, 513 tRNA loci from gtRNAdb (release 2009)^77^, 402 snoRNA, 1794 scRNA, 2007 snRNA loci as well as 722 other RNAs from UCSC annotation (hg19)^78^). The block groups were also compared to the 8,811 human ncRNA annotations from Rfam^79^. The block groups whose genomic coordinate overlap by at least one nucleotide with that of known annotations were designated as ‘annotated’ block groups (Supplementary Table S1, and S8). Similarly, block groups were compared with coordinates of exon, intron, 5′ UTR and 3′ UTR regions, all downloaded from UCSC^78^, and were annotated accordingly, if overlapping at >50% else designated as from intergenic region. If a block group overlaps to more than two regions then the region with maximum overlap is assigned to it.

### A pipeline for the identification of differential processing in RNA-seq data

**Figure 6:**
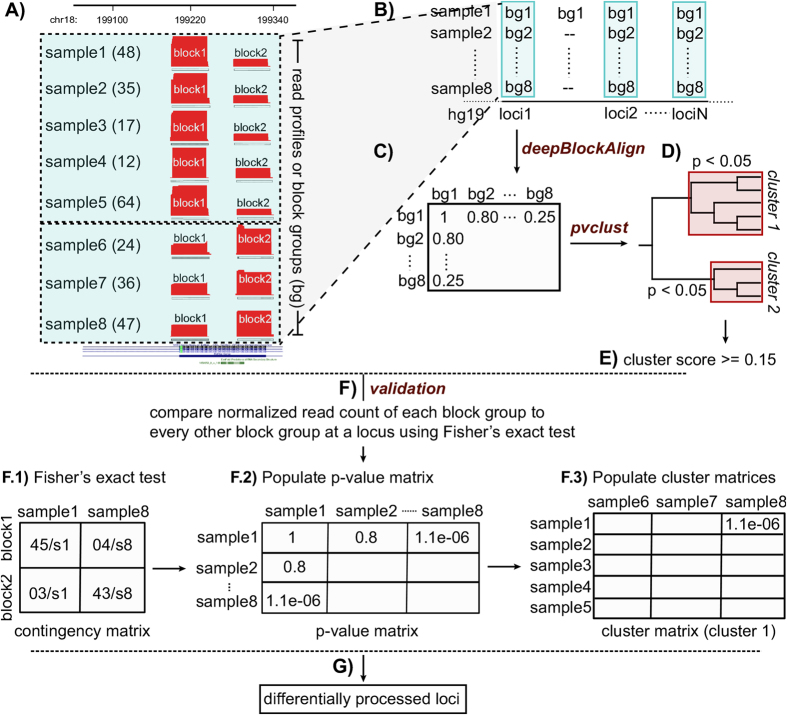
Various analysis steps to identify differential processing at a genomic locus. (**A**) Closely spaced mapped reads are grouped to define read profiles or block groups in each sample (see methods and Supplementary Fig. S1). A block group can consist of ≥1 block of reads. The numbers in the brackets represent the total read count within a block group. (**B**) We analyze all the genomic loci where a block group is observed in all the samples. (**C**) All the block groups (Supplementary Fig. S16) at a locus are aligned against each using deepBlockAlign^18^ to generate a square matrix of alignment scores (*S*). (**D**) A hierarchical cluster of block groups based on their alignment score is generated using pvclust, which also assigns a p-value indicating how strong the cluster is supported by the data^80^. We select all the clusters that have p-value < 0.05. (**E**) A cluster score (*X*) that measures how well separated the read profiles are within a cluster to read profiles outside the cluster is computed (Equation 2). We select all the loci having a cluster score of ≥0.15 as candidate differentially processed loci (DPL). (**F**) For each candidate DPL, we compare the normalized expression of block group from a sample to every other sample using Fisher’s exact test. (**F**.1) Specifically, we use m x 2 contingency matrix comprised of normalized expression of each block within the two block groups. Normalized expression of a block is computed by dividing it by a size factor (s), computed for each sample (Equation 4 **F**.2). A ‘p-value matrix’ is created that contains p-values computed from all vs. all comparison of normalized expression of block groups at a locus. (**F**.3) A ‘cluster matrix’ is created for each cluster identified in step-d. It consists of p-values computed from the comparison of normalized expression of block groups inside a cluster to those outside the cluster. (**G**) A locus is marked as differentially processed, if more than half of the samples in at least one ‘cluster matrix’ have significant p-value (<0.05) against all the samples outside the cluster.

In an earlier study^18^, we have developed a tool, deepBlockAlign for the alignment of two read profiles. deepBlockAlign normalizes the read counts by the total reads within a read profile followed by a two-tier strategy to align the read profiles (see Langenberger *et al.*^18^ for details). The alignment score (*S*) from deepBlockAlign ranges between 0 and 1 which suggests dissimilar and perfectly similar read profiles, respectively. In this study, we have built a pipeline integrating deepBlockAlign with appropriate preprocessing and validation steps to compare all the pairs of read profiles at a genomic locus derived from RNA-seq experiments performed on multiple cell lines or tissues (*N*). As input, the pipeline takes each genomic locus, *L* where a read profile is observed here, in all *N* cell lines (Fig. 6A). Next, a three step preprocessing procedure is followed for each locus (Supplementary Fig. S16). This procedure ensures that: a) block groups from all N cell lines have same number of read blocks; and, b) each constituent block contains its raw expression value that may have been altered due to a fixed cut-off of 10% (-minBlockHeight) which we took while defining the block groups using blockbuster. Firstly, all the non-overlapping (<90%) coordinates *C*_*l*_ at *l* = 1..*L* where a read block is observed in at least one cell line are determined. Secondly, for each coordinate *C*_*l*_, a single dummy read is placed followed by retrieval of all the reads that mapped at this coordinate from the BAM file (comprised of mapped reads). Thirdly, if all the block groups at a genomic locus have one read block, an adjacent dummy block is added that has an expression 10% relative to the expression of the parent block group. This is done due to the limitation of deepBlockAlign that requires at least two blocks of reads for meaningful comparison of read profiles or block groups.

After preprocessing, a series of analysis steps are performed to identify differentially processed loci (DPL), summarized in Fig. 6. Using deepBlockAlign, all the block groups at a locus (*l*) are aligned to each other to generate a square matrix of alignment scores (*S*), termed here as ‘alignment score matrix’ (Fig. 6B). Then based on this ‘alignment score matrix’, a hierarchical cluster of block groups is computed using the R package, pvclust^80^ (Fig. 6C). Using multiscale bootstrap resampling, pvclust computes a p-value for each constituent cluster in the hierarchical cluster indicating how strongly the cluster is supported by the data. We analyze all the clusters (*K*) with a p-value < 0.05. For the *k*th cluster, a score, *X*_*k*_, is computed as:


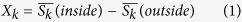


where 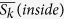 is the average alignment score between all the block groups within a cluster *k* and 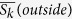 is the average alignment score between all the block groups inside cluster *k* and block groups outside the cluster *k*. The cluster score, *X*, at a locus is then computed as the average of score, *X*_*k*_ (Fig. 6D and Equation 2):


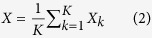


The cluster score, *X*, lies between 0 and 1, and should be maximized. The higher the cluster score, *X*, at a locus, the more dissimilar are the read profiles within a cluster in comparison to the rest of the read profiles outside the cluster. All the loci that have a cluster score, *X* ≥ 0.15 are selected as candidate differentially processed loci (Fig. 6E). The threshold of 0.15 for the cluster score is determined by comparing the frequency distribution of cluster scores obtained after analysis of read profiles from the Raz and ENCODE datasets (see Supplementary Methods for details). In order to select those loci that are differentially processed irrespective of the sequencing depth of the corresponding RNA-seq experiment, all the candidate loci are validated using Fisher’s exact test (Fig. 6F). More details about the validation step is described in the section below.

It should be noted that the pipeline can also be applied when the RNA-seq experiments have been replicated over the cell lines as it is the case for the ENCODE dataset analyzed in this study. However, differential processing analysis using all the replicates together requires two additional checks: a) Only those block groups that have both their replicates clustered together within the same cluster are used to compute the cluster score. This is done to ensure that only cell lines with consistent read profiles between the two replicates contribute to the cluster score; and, b) A locus is predicted as differentially processed only if at least one cluster has ≥4 block groups (two block groups per cell line).

### Validation of differential processing

To validate that the differential processing observed at a locus is not due to the sequencing artifact introduced by variable sequencing depth in different cell lines or tissues (*N*), we compare the normalized expression of block groups. Here, we have used the normalization method introduced by Anders *et al.*^37^ to identify differential expression between genes from two cell lines. Briefly, the method computes a size factor, *s*_j_ for each cell line, *j* so as to render read counts from these cell lines, which may have been sequenced to different depths, comparable. A size factor, *s*_*j*_ is computed by taking the median of the ratios of observed counts as


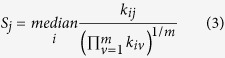


where *k*_*ij*_ is the raw read count for gene or read block *i* (in this case) in cell line *j*. The denominator of the expression above is interpreted as a pseudo-reference cell line obtained by taking the geometric mean across all cell lines, m. Next, we employ the size factors to normalize the expression of block groups at all the candidate loci. For each candidate locus (*l*) comprised of one or more clusters of distinct read profiles, the validation process is performed by computing the normalized expression (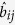) of each block *i*, within a block group, in cell line *j* as


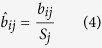


where b_*ij*_ is the raw read count for the block *i* in cell line *j*. Fisher’s exact test is then employed to compare the normalized expression of a block group from each cell line to every other block group at a locus (Fig. 6F.1) in order to create a ‘p-value matrix’ (Fig. 6F.2). For each cluster of block groups identified at a locus, a ‘cluster matrix’ is populated, that is comprised of p-values computed by comparison of normalized expression of block groups from cell lines inside a cluster to normalized expression of block groups from cell lines outside the cluster. A locus is then predicted as differentially processed, if most cell lines (>50%) in at least one ‘cluster matrix’ have significant p-value (<0.05) against all cell lines outside the cluster (Fig. 6F.3).

### Software availability

The Read Processing Analysis (RPA) pipeline used here was executed via a queue system on an x86_64 linux cluster consisting of 32 cores each having a memory of eight GB. The execution time was ten hours. We have made the software available via http://rth.dk/resources/rpa.

## Additional Information

**How to cite this article**: Pundhir, S. and Gorodkin, J. Differential and coherent processing patterns from small RNAs. *Sci. Rep.* **5**, 12062; doi: 10.1038/srep12062 (2015).

## Supplementary Material

Supplementary Information

## References

[b1] Windhager L. *et al.* Ultrashort and progressive 4sU-tagging reveals key characteristics of RNA processing at nucleotide resolution. Genome Res. 22, 2031–42 (2012). 22539649 10.1101/gr.131847.111 PMC3460197

[b2] Johnson J. M. *et al.* Genome-wide survey of human alternative pre-mRNA splicing with exon junction microarrays. Science (80-. ). 302, 2141–2144 (2003). 10.1126/science.1090100 14684825

[b3] Taft R. J., Pheasant M. & Mattick J. S. The relationship between non-protein-coding DNA and eukaryotic complexity. Bioessays 29, 288–99 (2007). 17295292 10.1002/bies.20544

[b4] Matlin A. J., Clark F. & Smith C. W. J. Understanding alternative splicing: towards a cellular code. Nat. Rev. Mol. Cell Biol. 6, 386–398 (2005). 15956978 10.1038/nrm1645

[b5] Lau N. C., Lim L. P., Weinstein E. G. & Bartel D. P. An abundant class of tiny RNAs with probable regulatory roles in Caenorhabditis elegans. Science (80-. ). 294, 858–862 (2001). 10.1126/science.1065062 11679671

[b6] Choudhuri S. Small noncoding RNAs: Biogenesis, function, and emerging significance in toxicology. J. Biochem. Mol. Toxicol. 24, 195–216 (2010). 20143452 10.1002/jbt.20325

[b7] Falaleeva M. & Stamm S. Processing of snoRNAs as a new source of regulatory non-coding RNAs: snoRNA fragments form a new class of functional RNAs. Bioessays 35, 46–54 (2013). 23180440 10.1002/bies.201200117 PMC3732821

[b8] Ellis J. D. *et al.* Short Article Tissue-Specific Alternative Splicing Remodels Protein-Protein Interaction Networks. Mol. Cell 46, 884–892 (2012). 22749401 10.1016/j.molcel.2012.05.037

[b9] Niemczyk M. *et al.* Imprinted chromatin around DIRAS3 regulates alternative splicing of GNG12-AS1, a long noncoding RNA. Am. J. Hum. Genet. 93, 224–235 (2013). 23871723 10.1016/j.ajhg.2013.06.010 PMC3738830

[b10] Wang Z., Jeon H. Y., Rigo F., Bennett C. F. & Krainer A. R. Manipulation of PK-M mutually exclusive alternative splicing by antisense oligonucleotides. Open Biol. 2, 120133–120133 (2012). 23155487 10.1098/rsob.120133 PMC3498831

[b11] Griffiths-Jones S., Hui J. H. L., Marco A. & Ronshaugen M. MicroRNA evolution by arm switching. EMBO Rep. 12, 172–7 (2011). 21212805 10.1038/embor.2010.191 PMC3049427

[b12] Kaczkowski B. *et al.* Structural profiles of human miRNA families from pairwise clustering. Bioinformatics 25, 291–294 (2009). 19059941 10.1093/bioinformatics/btn628

[b13] Kuchenbauer F. *et al.* Comprehensive analysis of mammalian miRNA* species and their role in myeloid cells. Blood 118, 3350–3358 (2011). 21628414 10.1182/blood-2010-10-312454

[b14] Jima D. D. *et al.* Deep sequencing of the small RNA transcriptome of normal and malignant human B cells identifies hundreds of novel microRNAs. Blood 116, e118–27 (2010). 20733160 10.1182/blood-2010-05-285403 PMC3012600

[b15] Zhang J. *et al.* Patterns of microRNA expression characterize stages of human B-cell differentiation. Blood 113, 4586–94 (2009). 19202128 10.1182/blood-2008-09-178186 PMC2680365

[b16] Cao G. *et al.* Differential expression of long non-coding RNAs in bleomycin-induced lung fibrosis. Int. J. Mol. Med. 32, 355–364 (2013). 23732278 10.3892/ijmm.2013.1404

[b17] Recio L. *et al.* Differential expression of long noncoding RNAs in the livers of female B6C3F1 mice exposed to the carcinogen furan. Toxicol. Sci. 135, 369–379 (2013). 23853263 10.1093/toxsci/kft153

[b18] Langenberger D. *et al.* deepBlockAlign: a tool for aligning RNA-seq profiles of read block patterns. Bioinformatics 28, 17–24 (2012). 22053076 10.1093/bioinformatics/btr598 PMC3244762

[b19] Findeiß S., Langenberger D., Stadler P. F. & Hoffmann S. Traces of post-transcriptional RNA modifications in deep sequencing data. Biol. Chem. 392, 305–313 (2011). 21345160 10.1515/BC.2011.043

[b20] Langenberger D., Bermudez-Santana C. I., Stadler P. F. & Hoffmann S. Identification and Classification of Small Rnas in Transcriptome Sequence Data. Biocomput. 2010 - Proc. Pacific Symp. 87, 80–87 (2010). 10.1142/9789814295291_0010 19908360

[b21] Friedländer M. R., MacKowiak S. D., Li N., Chen W. & Rajewsky N. MiRDeep2 accurately identifies known and hundreds of novel microRNA genes in seven animal clades. Nucleic Acids Res. 40, 37–52 (2012). 21911355 10.1093/nar/gkr688 PMC3245920

[b22] An J., Lai J., Lehman M. L. & Nelson C. C. miRDeep*: an integrated application tool for miRNA identification from RNA sequencing data. Nucleic Acids Res. 41, 727–737 (2013). 23221645 10.1093/nar/gks1187 PMC3553977

[b23] Kozomara A. & Griffiths-Jones S. miRBase: integrating microRNA annotation and deep-sequencing data. Nucleic Acids Res. 39, D152–D157 (2011). 21037258 10.1093/nar/gkq1027 PMC3013655

[b24] ENCODE Project Consortium and others. A User’s Guide to the Encyclopedia of DNA Elements (ENCODE). PLoS Biol 9, 21 (2011). 10.1371/journal.pbio.1001046 PMC3079585 21526222

[b25] Consortium, E. P. & others. An integrated encyclopedia of DNA elements in the human genome. Nature 489, 57–74 (2012). 22955616 10.1038/nature11247 PMC3439153

[b26] ’t Hoen P. A. C. *et al.* Reproducibility of high-throughput mRNA and small RNA sequencing across laboratories. Nat. Biotechnol. 31, 1015–22 (2013). 24037425 10.1038/nbt.2702

[b27] Marioni J. C., Mason C. E., Mane S. M., Stephens M. & Gilad Y. RNA-seq: an assessment of technical reproducibility and comparison with gene expression arrays. Genome Res. 18, 1509–1517 (2008). 18550803 10.1101/gr.079558.108 PMC2527709

[b28] Ro S., Park C., Young D., Sanders K. M. & Yan W. Tissue-dependent paired expression of miRNAs. Nucleic Acids Res. 35, 5944–5953 (2007). 17726050 10.1093/nar/gkm641 PMC2034466

[b29] Joglekar M., Patil D. & Joglekar V. The miR-30 family microRNAs confer epithelial phenotype to human pancreatic cells. Islets 1, 137–147 (2009). 21099261 10.4161/isl.1.2.9578

[b30] Zhou H. *et al.* Deep annotation of mouse iso-miR and iso-moR variation. Nucleic Acids Res. 40, 5864–5875 (2012). 22434881 10.1093/nar/gks247 PMC3401436

[b31] Chiang H. R. *et al.* Mammalian microRNAs: Experimental evaluation of novel and previously annotated genes. Genes Dev. 24, 992–1009 (2010). 20413612 10.1101/gad.1884710 PMC2867214

[b32] Hsieh L.-C. *et al.* Uncovering small RNA-mediated responses to phosphate deficiency in Arabidopsis by deep sequencing. Plant Physiol. 151, 2120–2132 (2009). 19854858 10.1104/pp.109.147280 PMC2785986

[b33] Breakfield N. W. *et al.* High-resolution experimental and computational profiling of tissue-specific known and novel miRNAs in Arabidopsis. Genome Res. 22, 163–176 (2012). 21940835 10.1101/gr.123547.111 PMC3246203

[b34] Li Z. *et al.* Extensive terminal and asymmetric processing of small RNAs from rRNAs, snoRNAs, snRNAs, and tRNAs. Nucleic Acids Res. 40, 6787–6799 (2012). 22492706 10.1093/nar/gks307 PMC3413118

[b35] Scott M. S. *et al.* Human box C/D snoRNA processing conservation across multiple cell types. Nucleic Acids Res. 40, 3676–3688 (2012). 22199253 10.1093/nar/gkr1233 PMC3333852

[b36] Hsu S. D. A. *et al.* MiRTarBase: A database curates experimentally validated microRNA-target interactions. Nucleic Acids Res. 39, D163–D169 (2011). 21071411 10.1093/nar/gkq1107 PMC3013699

[b37] Anders S. & Huber W. Differential expression analysis for sequence count data. Genome Biol. 11, R106 (2010). 20979621 10.1186/gb-2010-11-10-r106 PMC3218662

[b38] Li H. *et al.* The sequence alignment/map format and SAMtools. Bioinformatics 25, 2078–2079 (2009). 19505943 10.1093/bioinformatics/btp352 PMC2723002

[b39] Dohm J. C., Lottaz C., Borodina T. & Himmelbauer H. Substantial biases in ultra-short read data sets from high-throughput DNA sequencing. Nucleic Acids Res. 36, e105–e105 (2008). 18660515 10.1093/nar/gkn425 PMC2532726

[b40] Zheng W., Chung L. M. & Zhao H. Bias detection and correction in RNA-Sequencing data. BMC Bioinformatics 12, 290 (2011). 21771300 10.1186/1471-2105-12-290 PMC3149584

[b41] Taft R. J. *et al.* Small RNAs derived from snoRNAs. RNA 15, 1233–40 (2009). 19474147 10.1261/rna.1528909 PMC2704076

[b42] Brameier M., Herwig A., Reinhardt R., Walter L. & Gruber J. Human box C/D snoRNAs with miRNA like functions: Expanding the range of regulatory RNAs. Nucleic Acids Res. 39, 675–686 (2011). 20846955 10.1093/nar/gkq776 PMC3025573

[b43] Haussecker D. *et al.* Human tRNA-derived small RNAs in the global regulation of RNA silencing. RNA 16, 673–95 (2010). 20181738 10.1261/rna.2000810 PMC2844617

[b44] Lee Y. S., Shibata Y., Malhotra A. & Dutta A. A novel class of small RNAs: tRNA-derived RNA fragments (tRFs). Genes Dev. 23, 2639–49 (2009). 19933153 10.1101/gad.1837609 PMC2779758

[b45] Cole C. *et al.* Filtering of deep sequencing data reveals the existence of abundant Dicer-dependent small RNAs derived from tRNAs. RNA 15, 2147–60 (2009). 19850906 10.1261/rna.1738409 PMC2779667

[b46] Smalheiser N. R., Lugli G., Thimmapuram J., Cook E. H. & Larson J. Endogenous siRNAs and noncoding RNA-derived small RNAs are expressed in adult mouse hippocampus and are up-regulated in olfactory discrimination training. RNA 17, 166–181 (2011). 21045079 10.1261/rna.2123811 PMC3004058

[b47] Thompson D. M., Lu C., Green P. J. & Parker R. tRNA cleavage is a conserved response to oxidative stress in eukaryotes. RNA 14, 2095–2103 (2008). 18719243 10.1261/rna.1232808 PMC2553748

[b48] Kishore S. *et al.* The snoRNA MBII-52 (SNORD 115) is processed into smaller RNAs and regulates alternative splicing. Hum. Mol. Genet. 19, 1153–1164 (2010). 20053671 10.1093/hmg/ddp585 PMC2838533

[b49] Ernst J. & Kellis M. Discovery and characterization of chromatin states for systematic annotation of the human genome. Nat. Biotechnol. 28, 817–825 (2010). 20657582 10.1038/nbt.1662 PMC2919626

[b50] Hoffman M. M. *et al.* Unsupervised pattern discovery in human chromatin structure through genomic segmentation. Nat. Methods 9, 473–476 (2012). 22426492 10.1038/nmeth.1937 PMC3340533

[b51] Torarinsson E. *et al.* Comparative genomics beyond sequence-based alignments: RNA structures in the ENCODE regions. Genome Res 18, 242–251 (2008). 18096747 10.1101/gr.6887408 PMC2203622

[b52] Zamudio J. R., Kelly T. J. & Sharp P. A. Argonaute-bound small RNAs from promoter-proximal RNA polymerase II. Cell 156, 920–934 (2014). 24581493 10.1016/j.cell.2014.01.041 PMC4111103

[b53] Seila A. C. *et al.* Divergent transcription from active promoters. Science (80-. ). 322, 1849–1851 (2008). 10.1126/science.1162253 PMC2692996 19056940

[b54] Taft R. J. *et al.* Tiny RNAs associated with transcription start sites in animals. Nat. Genet. 41, 572–578 (2009). 19377478 10.1038/ng.312

[b55] Fejes-Toth K. *et al.* Post-transcriptional processing generates a diversity of 5-modified long and short RNAs. Nature 457, 1028–1032 (2009). 19169241 10.1038/nature07759 PMC2719882

[b56] Valen E. *et al.* Biogenic mechanisms and utilization of small RNAs derived from human protein-coding genes. Nat. Publ. Gr. 18, 1075–1082 (2011). 10.1038/nsmb.2091 21822281

[b57] Ntini E. *et al.* Polyadenylation site–induced decay of upstream transcripts enforces promoter directionality. Nat. Struct. Mol. Biol. 20, 923–928 (2013). 23851456 10.1038/nsmb.2640

[b58] Core L. J. *et al.* Analysis of transcription start sites from nascent RNA supports a unified architecture of mammalian promoters and enhancers. Nat Genet 46, 1311–1320 (2014). 25383968 10.1038/ng.3142 PMC4254663

[b59] Derrien T. *et al.* The GENCODE v7 catalog of human long noncoding RNAs: analysis of their gene structure, evolution, and expression. Genome Res. 22, 1775–89 (2012). 22955988 10.1101/gr.132159.111 PMC3431493

[b60] Thurman R., Rynes E. & Humbert R. The accessible chromatin landscape of the human genome. Nature 489, 75–82 (2012). 22955617 10.1038/nature11232 PMC3721348

[b61] Bartel D. P. MicroRNAs: Target Recognition and Regulatory Functions. Cell 136, 215–233 (2009). 19167326 10.1016/j.cell.2009.01.002 PMC3794896

[b62] Hennig G., Lowrick O., Birchmeier W. & Behrens J. Mechanisms identified in the transcriptional control of epithelial gene expression. J. Biol. Chem. 271, 595–602 (1996). 8550625 10.1074/jbc.271.1.595

[b63] Higashikawa K. *et al.* Snail-induced down-regulation of DeltaNp63alpha acquires invasive phenotype of human squamous cell carcinoma. Cancer Res. 67, 9207–9213 (2007). 17909026 10.1158/0008-5472.CAN-07-0932

[b64] Ender C. *et al.* A Human snoRNA with MicroRNA-Like Functions. Mol. Cell 32, 519–528 (2008). 19026782 10.1016/j.molcel.2008.10.017

[b65] Emani S., Zhang J., Guo L., Guo H. & Kuo P. C. RNA Stability regulates differential expression of the metastasis protein, osteopontin, in hepatocellular cancer. Surgery 143, 803–812 (2008). 18549897 10.1016/j.surg.2008.02.005 PMC2494577

[b66] Jung C.-H., Hansen M. A., Makunin I. V., Korbie D. J. & Mattick J. S. Identification of novel non-coding RNAs using profiles of short sequence reads from next generation sequencing data. BMC Genomics 11, 77 (2010). 20113528 10.1186/1471-2164-11-77 PMC2825236

[b67] Erhard F. & Zimmer R. Classification of ncRNAs using position and size information in deep sequencing data. Bioinformatics 26, i426–i432 (2010). 20823303 10.1093/bioinformatics/btq363 PMC2935403

[b68] Castle J. C. *et al.* Digital genome-wide ncRNA expression, including SnoRNAs, across 11 human tissues using polyA-neutral amplification. PLoS One 5, e11779 (2010). 20668672 10.1371/journal.pone.0011779 PMC2909899

[b69] Pruitt K. D., Tatusova T. & Maglott D. R. NCBI reference sequences (RefSeq): A curated non-redundant sequence database of genomes, transcripts and proteins. Nucleic Acids Res. 35, D61–65 (2007). 17130148 10.1093/nar/gkl842 PMC1716718

[b70] Hubbard T. *et al.* The Ensembl genome database project. Nucleic Acids Res. 30, 38–41 (2002). 11752248 10.1093/nar/30.1.38 PMC99161

[b71] Barrett T. *et al.* NCBI GEO: Archive for functional genomics data sets - Update. Nucleic Acids Res. 41, D991–995 (2013). 23193258 10.1093/nar/gks1193 PMC3531084

[b72] Raz T. *et al.* Protocol dependence of sequencing-based gene expression measurements. PLoS One 6, e19287 (2011). 21573114 10.1371/journal.pone.0019287 PMC3089619

[b73] Goecks J., Nekrutenko A., Taylor J. & Team T. G. Galaxy: a comprehensive approach for supporting accessible, reproducible, and transparent computational research in the life sciences. Genome Biol 11, R86 (2010). 20738864 10.1186/gb-2010-11-8-r86 PMC2945788

[b74] Karolchik D. *et al.* The UCSC genome browser database. Nucleic Acids Res. 31, 51–54 (2003). 12519945 10.1093/nar/gkg129 PMC165576

[b75] Hoffmann S. *et al.* Fast mapping of short sequences with mismatches, insertions and deletions using index structures. PLoS Comp. Biol. 5, e1000502 (2009). 10.1371/journal.pcbi.1000502 PMC2730575 19750212

[b76] Langenberger D. *et al.* Evidence for human microRNA-offset RNAs in small RNA sequencing data. Bioinformatics 25, 2298–2301 (2009). 19584066 10.1093/bioinformatics/btp419

[b77] Chan P. P. & Lowe T. M. GtRNAdb: a database of transfer RNA genes detected in genomic sequence. Nucleic Acids Res. 37, D93–D97 (2009). 18984615 10.1093/nar/gkn787 PMC2686519

[b78] Karolchik D. *et al.* The UCSC Table Browser data retrieval tool. Nucleic Acids Res. 32, D493–D496 (2004). 14681465 10.1093/nar/gkh103 PMC308837

[b79] Gardner P. P. *et al.* Rfam: Wikipedia, clans and the decimal release. Nucleic Acids Res. 39, D141–145 (2011). 21062808 10.1093/nar/gkq1129 PMC3013711

[b80] Suzuki R. & Shimodaira H. Pvclust: an R package for assessing the uncertainty in hierarchical clustering. Bioinformatics 22, 1540–1542 (2006). 16595560 10.1093/bioinformatics/btl117

