# Supplementary material for: Differential and coherent processing patterns from small RNAs
Source: Sci Rep. 2015 Jul 13;5:12062. doi: 10.1038/srep12062 (PMC4499813; doi:10.1038/srep12062)
Supplement: Supplementary Information [file srep12062-s1.pdf]

Differential and coherent processing patterns from small RNAs

Sachin Pundhir, Jan Gorodkin*
Center for non-coding RNA in Technology and Health, IKVH, University of Copenhagen, Grønnegårdsvej 3, 1870 Frederiksberg C, Denmark.
*Correspondence: gorodkin@rth.dk

Supplementary methods 2
Supplementary results 5
Supplementary figures 10
Supplementary tables 27
Supplementary references 36
Graphical plots of 97 differentially processed loci 38
Graphical plots of 158 annotated coherently processed loci 39
Graphical plots of 195 unannotated coherently processed loci 40

Supplementary Methods

Bionomial test for the enrichment analysis of 353 coherently processed loci (CPL) at 5' UTR, 3' UTR, Exon and Intron

The *p*-value is computed in a similar way as done in[1]. The number out of 353 CPL (M) with which the CPL overlaps with a genomic region (5' UTR, 3' UTR, Exon and Intron) is compared with the frequency of overlaps that can be expected under the null model where each CPL is a dart thrown randomly onto the genome. If a genomic region covers a fraction *P* of the human genome (3 billion bases), then under the simple binomial model: each of the 353 CPL has probability *P* of overlapping a genomic locus. For 353 CPL, the expected number of overlapping CPL is $\mu = 353 * P$, with a standard deviation

$$\sigma = \sqrt{N * P * (1 - P)}$$

We then calculate the *p*-value using the normal approximation of the binomial distribution, `pnorm` function in R where;

P[X $>$ x] = pnorm(M, μ, σ, lower.tail=F)

Binomial test for the enrichment analysis of 195 unannotated coherently processed loci (CPL) at the seven distinct chromatin states

The *p*-value is computed in a similar way as done in[1]. The number out of 195 unannotated CPL (M) with which the CPL overlaps with a chromatin state is compared with the frequency of overlaps that can be expected under the null model where each CPL is a dart thrown randomly onto the genome. If a chromatin state covers a fraction *P* of the human genome (3 billion bases), then under the simple binomial model: each of the 195 CPL has probability *P* of overlapping a chromatin state. For 195 CPL, the expected number of overlapping CPL is $\mu = 195 * P$, with a standard deviation

$$\sigma = \sqrt{N * P * (1 - P)}$$

We then calculate the *p*-value using the normal approximation of the binomial distribution, `pnorm` function in R where;

P[X $>$ x] = pnorm(M, μ, σ, lower.tail=F)

ENCODE genome segmentation tracks

We used the ENCODE genome segmentation tracks available from the UCSC table browser[2,3] to study the enrichment of coherently processed loci at these genome segments. In these tracks, human genome has been divided into seven distinct chromatin states: a) Transcription start site (TSS) including promoter region, b) Promoter flanking (PF) region, c) Enhancer (E) region, d) Weak enhancer (WE) or open chromatin *cis*-regulatory element, e) CCCTC-Binding factor (CTCF) enriched element, f) Transcribed region (T); and, g) Repressed (R) or low activity regions. To determine these states, ChIP-seq data for eight chromatin marks (H3K27ac, H3K27me3, H3K36me3, H3K4me1, H3K4me2, H3K4me3, H3K9ac, H4K20me1 and an input control signal), the CTCF transcription factor marks, two DNase-seq assays and a FAIRE-seq assay was used to train two machine learning methods, ChromHMM[4] and Segway[5], respectively. Both of these methods were then used to computationally predict the seven genome segments across six cell-lines (GM12878, H1-hESC, HeLa-S3, HepG2, HUVEC and K562). In this study, we have used the consensus for the predictions of the seven chromatin states from the two computational methods on each of the six cell lines.

Statistical tests

Significant difference between two distributions was assessed using the Kolmogorov-Smirnov test. This test is non-parametric statistical hypothesis test that compare two distributions based on the ranks of observations and, thus, does not require any assumptions on the type of distribution. Significance of enrichment was evaluated using Fisher's exact test, which test for a significant association between two different types of classification.

Computation of threshold value for the cluster score

To determine the threshold for the cluster score, X, we compared the frequency distribution of cluster scores obtained after differential processing analysis of the Raz and ENCODE datasets. For the ENCODE dataset, we analyzed both sets of biological replicates separately as well as by combining them together. Since, in the Raz dataset, we are comparing the read profiles across six replicates of the same cell line (liver), we expect a lower limit to maximum cluster score in comparison to that for the ENCODE dataset, where we are comparing the read profiles across nine different human cell lines. Furthermore, the analysis of the Raz

dataset can also provide an estimate of cluster score that can be obtained just due to variation in the read profiles between the replicates. We observed distinct distributions of cluster score from the two datasets (Figure S2). While the distribution of cluster scores computed using the Raz dataset is unimodal, a bimodal distribution is observed for all the three distributions of cluster scores computed using the ENCODE dataset. Based on the score distributions, we chose an empirical cut-off of $\geq$0.15 to define a locus as differentially processed. Furthermore, at a cut-off of 0.15, we predicted 38 (1%) out of 3,351 loci as differentially processed in the Raz dataset that is much less in comparison to 97 (14%) out of 701 loci predicted to be differentially processed in the ENCODE dataset (combined analysis of replicates).

Supplementary Results

Reproducibility of read profiles

To gain insight into the extent of reproducibility between read profiles from the replicates of RNA-seq experiments performed in the same as well as different laboratories, we analyzed six arbitrary selected short RNA-seq experiments downloaded from the GEO[6]. These experiments have been performed on four different tissues in same as well as different laboratories (Supplementary Table S5). Specifically, we computed alignment scores, using `deepBlockAlign`[7], between read profiles from pairs of experiments arranged in four combinations (Supplementary Table S5):

- two experiments performed on the same tissue (biological replicates) in the same laboratory,
- two experiments performed on the different tissues in the same laboratory,
- two experiments performed on the same tissue in the different laboratories; and,
- two experiments performed on the different tissues in the different laboratories

We observed a significantly higher proportion of read profiles derived from the same tissues (both from the same and different laboratories) to have higher alignment scores in comparison to those read profiles derived from different tissues (Supplementary Figure S3). The higher the alignment score, the more similar are the read profiles between the two experiments. Specifically, 95% of the read profiles compared between two experiments, performed on the same tissue in either same or different laboratory, obtained an alignment score above the empirical cutoff of $\geq$0.6 and $\geq$0.55, respectively. In contrast, a significantly lower percentage of 75% and 66% read profiles showed an alignment score of $\geq$0.6 and $\geq$0.55 between experiments performed on different tissues in the same and different laboratory, respectively (p-value$<$0.001, Fisher's exact test; Supplementary Table S5).

To complement, we also compared the consistency between the read profiles from the same tissue to those from different tissues, using both the biological replicates of the ENCODE dataset. We note that the read profiles from two different tissues at a locus can also be very similar[8], however, the alignment scores between

them can provide a conservative estimate of the consistency that can be observed between two randomly chosen read profiles. As shown in Supplementary Figure S5D, the `deepBlockAlign` scores between same tissue read profiles are significantly higher in comparison to scores between read profiles from different tissues (*p*-value=7.9e-45, Kolmogorov-Smirnov test). Thus, our results suggest that read profile of a transcript is a reproducible phenomenon that by being more consistent between replicates of the same tissue often represents the processing mechanism of the host transcript. Also, the reproducibility between read profiles was not observed to be dependent upon their expression in the two replicates (Figure S4).

Reproducibility of differential processing

Owing to the reproducibility of read profiles, we observed high correlation (R^2) between the cluster scores obtained from the independent and combined analysis of the replicates. Specifically, we compared the cluster scores obtained after the analysis of the two replicates (replicate 1 or 2 of nine cell lines) from the ENCODE dataset independently against combining them together (Supplementary Figure S5A, B and C). Most DPL (marked by a red circle) were observed to have consistently high cluster scores in both set of comparisons. For the agreement between the predictions, we observed a sensitivity, specificity and Matthew's Correlation Coefficient (MCC) of 0.71, 0.92 and 0.59, respectively, for DPL identified on the combined and the independent analysis of replicate 1. Similar sensitivity, specificity and MCC of 0.71, 0.90 and 0.55, respectively were observed on the combined and the independent analysis of replicate 2 (Supplementary Table S6).

To compute the above mentioned performance measures, we compared the number of DPL identified upon the combined and the independent analysis of the replicates by creating two confusion or contingency matrices corresponding to the two sets of comparisons (combined against independent analysis of replicate 1 and combined against independent analysis of replicate 2). A confusion matrix is comprised of four numbers:

- the number of DPL identified in both the combined and the independent analysis as True Positives (TP),
- the number of DPL identified in the combined analysis only as False Negatives (FN),
- the number of DPL identified in the independent analysis only as False Positives (FP); and,

- the number of non-DPL identified in both the combined and the independent analysis as True Negatives (TN).

We considered the DPL identified on the combined analysis as TP due to the stringent criteria of requiring both the biological replicates of a read profile in the same cluster during the differential processing analysis (see methods). This requirement makes the results from the combined analysis more confident in comparison to those obtained on the independent analysis. Based on the two confusion matrices, we computed the sensitivity, specificity and mathew's correlation coefficient (MCC).

As shown in Supplementary Figure S5C, for some loci, we also observed inconsistent cluster scores characterized by the cluster score of $\geq$0.15 in only one of the two biological replicates. In total, out of 171 DPL predicted in either of the two replicates, 73 showed consistent cluster score and the remaining 98 were observed with inconsistent cluster scores. Figure 1D presents a representative example of a locus where we observed inconsistent cluster scores between the two replicates. The example illustrates a DPL encoding for a snoRNA and is characterized by two distinct set of read profiles, one having most of the expression from the 3' end and another having most of the expression from the 5' end. Almost all the read profiles are well consistent between the two biological replicates. However, due to the variable read profile in skin, inconsistent cluster scores of 0.19 and 0.14 were observed for replicate 1 and replicate 2, respectively. Also, the read profile from skin is inconsistently clustered (marked by different color of the tree branches) in the two replicates. Since our differential processing pipeline in its essence compares the relative expression and position of reads within a read profile, presence or absence of a few reads due to low sequencing depth in a cell line or biological variation between the replicates can lead to such discrepancies.

Indeed, the alignment scores between read profiles from the two biological replicates were significantly (p-value=0.005, Kolmogorov-Smirnov test; Supplementary Figure S5D) higher for 73 loci where consistent cluster scores are observed in comparison to 98 loci exhibiting inconsistent cluster scores. Similarly, we observed significantly higher alignment scores for read profiles that clustered consistently in comparison to those that clustered inconsistently (p-value=6.8e-15, Kolmogorov-Smirnov test; Supplementary Figure S5D) between the two biological replicates. This suggests that high variation in some read profiles between the two replicates can be attributed as the primary reason for inconsistency in both the cluster scores and clustering. We note that although variability in read profile leads to inconsistency in both cluster scores and clustering, no such clear relation is observed between cluster scores and clustering i.e. loci with consistent clusters

can still exhibit inconsistent cluster scores between the replicates and vice-versa (Supplementary Figure S6).

The effect of local sequence context on read profiles

Recent work have studied the effects of local sequencing context like GC% on the reproducibility of RNA-seq experiments[9,10,11]. The GC content and various dinucleotide frequencies related to GC content have been shown to influence the evenness of transcript coverage[12,9]. We investigated the effect of local sequence context on DP by comparing the GC% and frequencies of mono-, di- and trinucleotides between 97 DPL and 23,047 background loci in the ENCODE dataset. A background locus is defined as a locus where a block group is observed in at least one replicate of the nine cell lines.

In supplementary figure S7 we show the density distribution of GC% between the two sets of loci. The two density distributions (differentially processed and background) were compared individually for each of the six bins of block group length (Supplementary Figure S7A-L) using Kolmogorov-Smirnov test, p-value$<$0.05. Similarly, we compared the density distribution of the 4 mono-, 16 di- and 64 tri-nucleotides between the background and DPL (Supplementary Figure S8, S9 and S10). The 4 mono-, 16 di- and 64 tri- nucleotides are based on the requirement that each of these combinations can have one out of the four nucleotides (A, T, G and C) at each position. For both GC% and frequencies of mono-, di- and trinucleotides, we observed no significant difference (Kolmogorov-Smirnov test, p-value$<$0.05) in the density distribution of the nucleotides between DP and background loci.

Next, we measured any bias in the read count between the 5' and 3' ends of the block groups. We retrieved all the block groups from the ENCODE dataset and divided each block group into two halves, first starting from the 5' end to the mid point and second starting from the mid point till the 3' end. The mean read count per nucleotide position is computed for each half of the block groups. We observed no significant difference in the density distribution of the mean read count (log) between the two ends of the block groups (Kolmogorov-Smirnov test, p-value$<$0.05 and Supplementary Figure S11).

Another form of bias has been suggested as the protocol-specific sequence bias where the read count at a particular nucleotide position depends on the sequencing protocol[13]. The authors in[13] have developed a bayesian network to correct for these biases, primarily in mRNA-seq protocols. Here, we used this method to correct for the bias at the 701 genomic loci analyzed in this study and measured

the correlation between the cluster scores obtained before and after correcting for sequence bias. We observed a high spearman's rank correlation coefficent (R^2) of 0.8 between the cluster scores (Supplementary Figure S12). Furthermore, 68 and 23 out of the 97 DPL identified previously, showed a cluster score higher than or close to 0.15 (threshold for identifying differentially processed loci), respectively. In conclusion, none of the analyses suggest that various sequence features affect the transcript coverage for the DPL candidates.

Effect of differential expression on differential processing

We analyzed 97 DPL identified on combined analysis of the ENCODE dataset for differential expression using R package, `DESeq`. `DESeq` is a method based on negative binomial distribution to identify differential expression between multiple replicates of two experimental conditions[14]. For each DPL with one or more clusters, we compared the absolute read count of block groups within a cluster with the rest of the block groups. We consider the block groups within a cluster as replicates from one experimental condition and the rest of the block groups as replicates from another experimental condition. `DESeq` computes a *p*-value and the fold-change in the expression that suggests the extent of differential expression between the two experimental conditions i.e. expression of block groups within a cluster and outside the cluster. Of the 97 loci, we observed only one locus where read profiles within a cluster are differentially expressed from those outside the cluster (*p*-value$<$0.01, adjusted by Benjamini-Hochberg procedure). This suggests that the DP observed at these loci can not be attributed to the difference in absolute or relative expression between the cell lines exhibiting distinct profiles.

Supplementary Figures

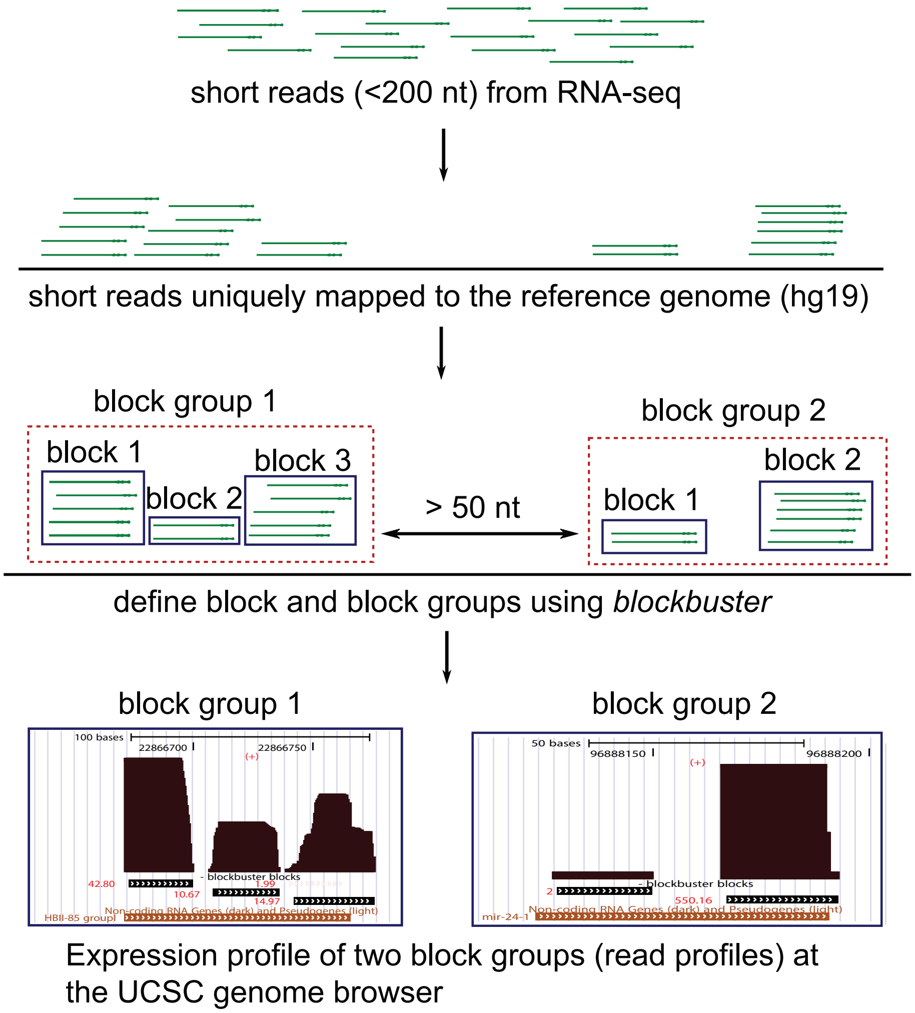

Supplementary Figure S1: Processing of mapped reads to define block groups or read profiles. Block groups are defined using `blockbuster`[15], which assigns two reads to the same locus when they are separated by <50 nt, followed by diving consecutive reads at each locus into read blocks. Thus, a block group essentially contains one or more read blocks and each of its position represents the number of mapped reads, which ultimately defines its expression profile (read profile).

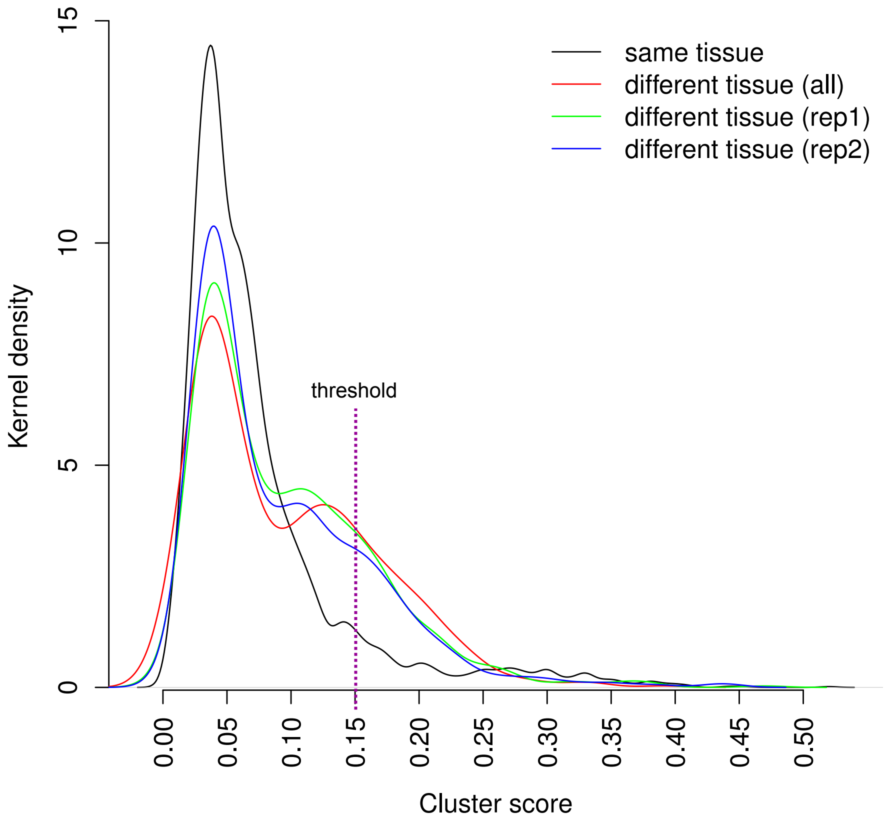

Supplementary Figure S2: Density distribution of the cluster scores (S) obtained after differential processing analysis of Raz (same cell line) and ENCODE (different cell lines) dataset. As expected, we observed a lower density of loci with high cluster scores in Raz in comparison to ENCODE dataset. Similar bimodal distributions were observed for both combined (all) and independent (rep1 and rep2) analysis of replicates from ENCODE dataset. Based on the distributions, we chose an empirical cut-off of 0.15 to predict a locus as differentially processed.

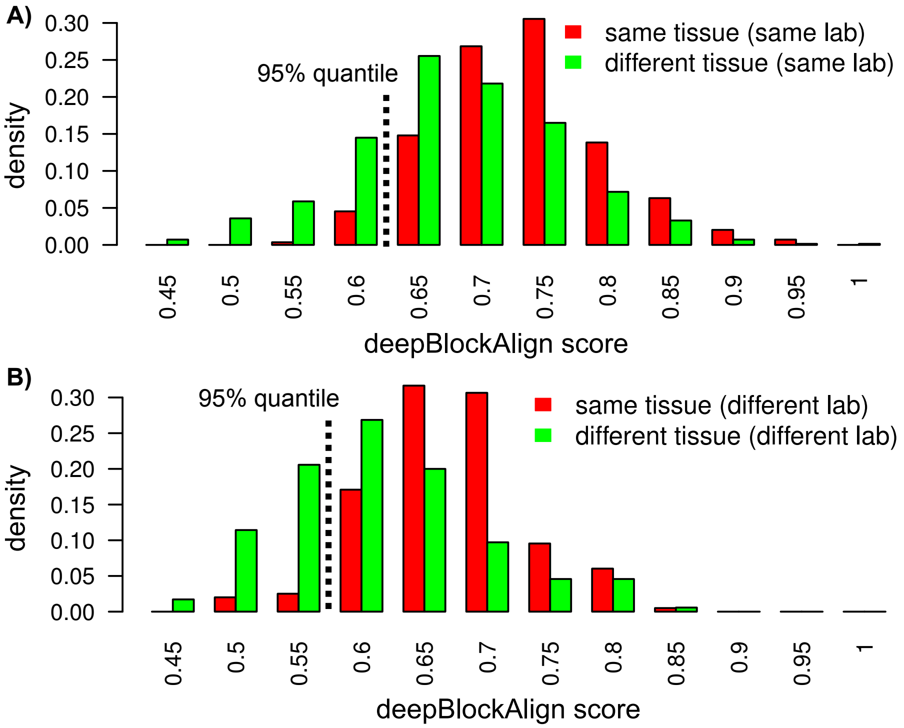

Supplementary Figure S3: The density distribution of the `deepBlockAlign` alignment scores obtained after comparison of read profiles between two short RNA-seq experiments. A) alignment scores between read profiles from experiments performed on two biological replicates of blood in the same laboratory (same tissue and lab) and from experiments performed on blood and brain in the same laboratory (different tissue and same lab). Most ($\sim$95%, black vertical line) alignment scores from former distribution are $\geq$0.6. B) alignment score between read profiles from experiments performed on testes samples in two different laboratories (same tissue and different lab) and from experiments performed on testes and germinal center B-cells in two different laboratories (different tissue and different lab). Most ($\sim$95%, black vertical line) alignment score from former distribution are $\geq$0.55. Both the distributions suggest that we can expect most pair of read profiles derived from experiments performed on same tissues in same as well as different laboratories and that have variability in the arrangement of mapped reads imposed due to biological or technical variation to have a higher alignment score in comparison to the alignment score between read profiles derived from different tissues.

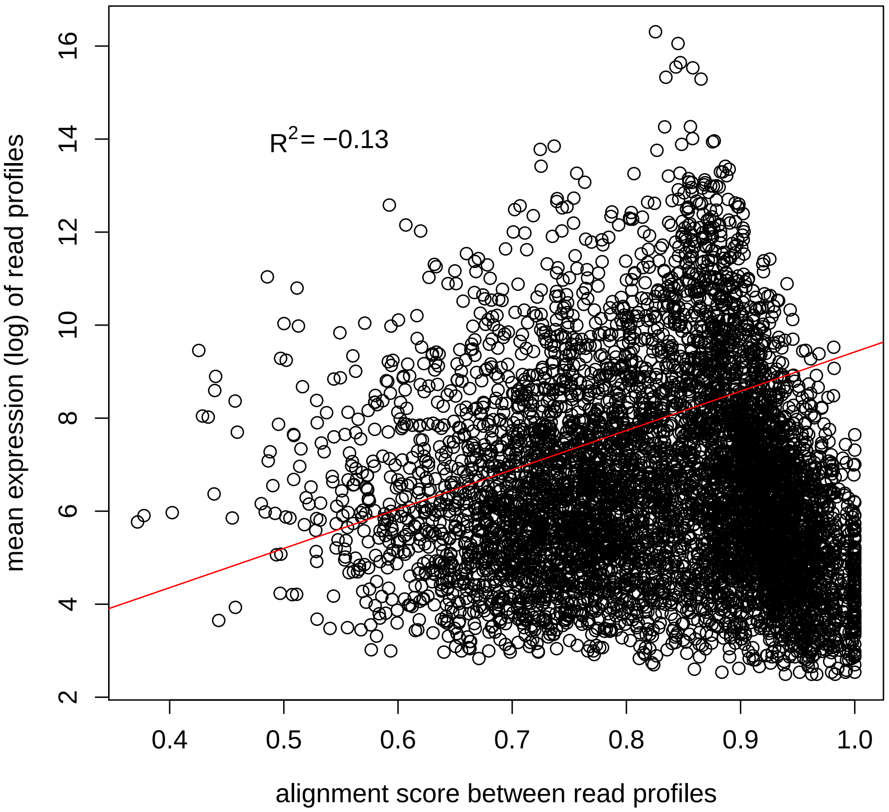

Supplementary Figure S4: The relation between the extent of reproducibility in read profiles and their expression from the two biological replicates of ENCODE dataset. The extent of reproducibility is measured between two read profiles (replicate 1 and 2) from the same cell line at 701 genomic loci using `deepBlockAlign`. `deepBlockAlign` computes an alignment score between two read profiles[7]. A high score suggests more similarity between two read profiles (x-axis). No positive correlation (Spearman's rank correlation coefficient, R^2 = -0.13) is observed between the reproducibility of read profiles and their expression (mean of the expression from the two replicates; y-axis), suggesting it being independent of the amount of reads constituting a read profile.

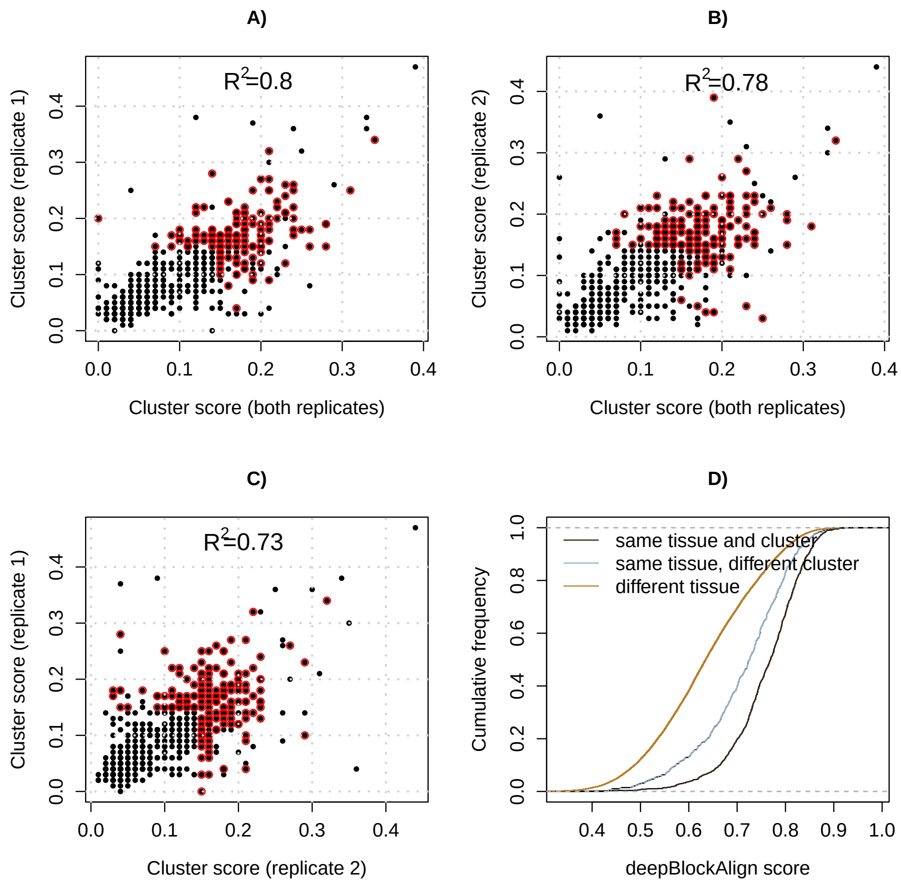

Supplementary Figure S5: Reproducibility in differential processinng measured upon independent and combined analysis of the two biological replicates from the ENCODE dataset. A) high correlation of 0.8 (Spearman's rank correlation coefficient, R^2, *p*-value=3.2e-158) is observed between the cluster scores from independent analysis of replicate 1 and combined analysis of both replicates (all). Similar high correlation of 0.78 (*p*-value=3.9e-147) and 0.73 (*p*-value=4.6e-116) is also observed from analysis between replicate 2 and all (B) and replicate 1 and replicate 2 (C), respectively. Cluster scores for differentially processed loci (DPL) are marked in red. D) The cumulative frequency distribution of alignment scores between read profiles from the two biological replicates, computed using `deepBlockAlign`. The alignment score between read profiles from same cell line is significantly higher in comparison to the scores between read profiles from two different cell lines (*p*-value=7.9e-45, Kolmogorov-Smirnov test). Also, the alignment score between read profiles that cluster consistently is significantly higher as compared to those read profiles which clustered inconsistently between the two biological replicates (*p*-value=6.8e-15, Kolmogorov-Smirnov test). This suggests that although read profiles from the two replicates of same cell line are significantly more coherent in comparison to the read profiles from different cell line, there exists a level of biological variation between the read profiles from the two replicates that leads to inconsistency in cluster scores. Here, the alignment score between read profiles from two different cell lines provide a conservative estimate of alignment score that can be expected between two random read profiles.

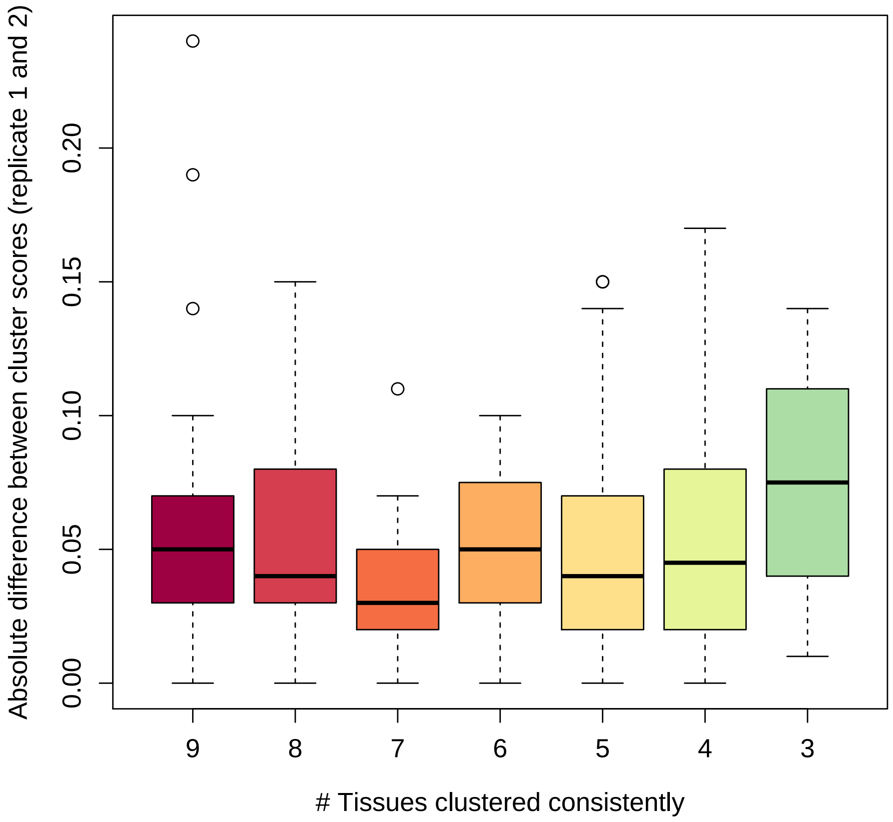

Supplementary Figure S6: Relation between number of cell lines that clustered consistently at a locus with the absolute difference in cluster score between the two biological replicates for 171 differentially processed loci identified either in replicate 1 or replicate 2 of ENCODE dataset. No clear relation is observed between inconsistent clustering and cluster scores as evident from the observation that loci with consistent clusters can also exhibit inconsistent cluster scores and vice-versa.

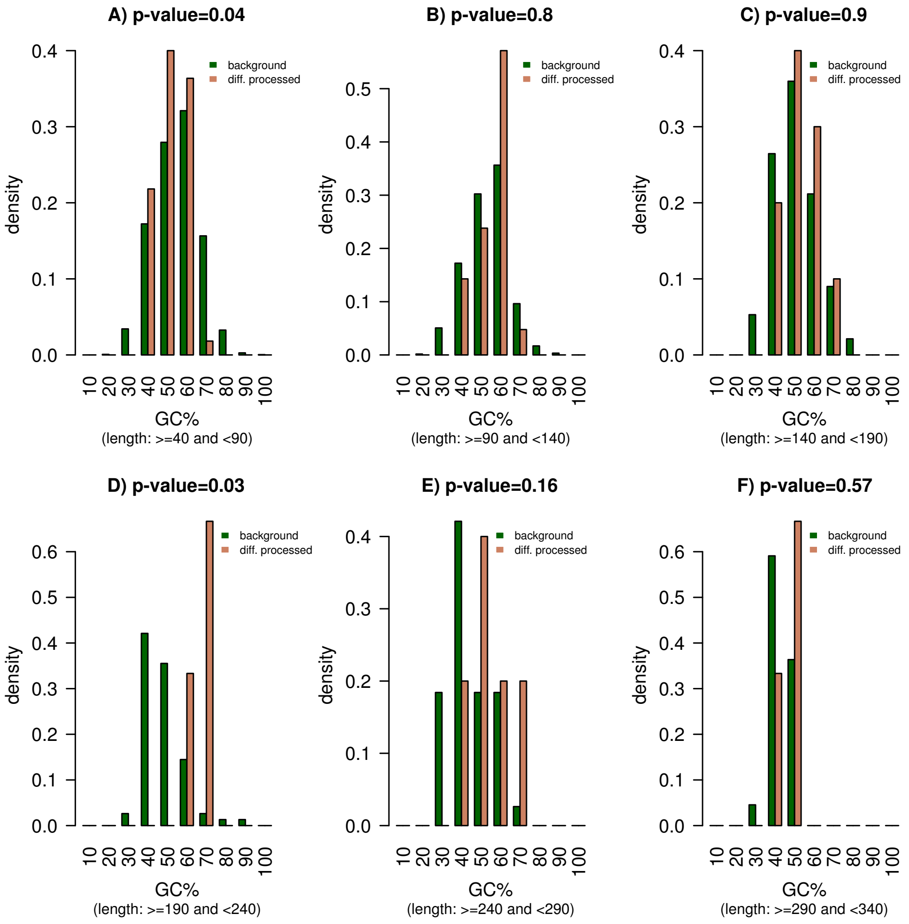

Supplementary Figure S7: Density distribution of GC% between the loci where expression is observed in atleast one of the N cell lines (background) and differentially processed loci. The two density distributions were compared individually for each of the 12 bins of block group length (A-L) using Kolmogorov-Smirnov test, *p*-value < 0.01. We observed no significant difference in the GC% between the differentially processed and background loci suggesting that differential processing identified at a locus is not effected by the technical artifacts imposed during the sequencing process by the GC content of a transcript.

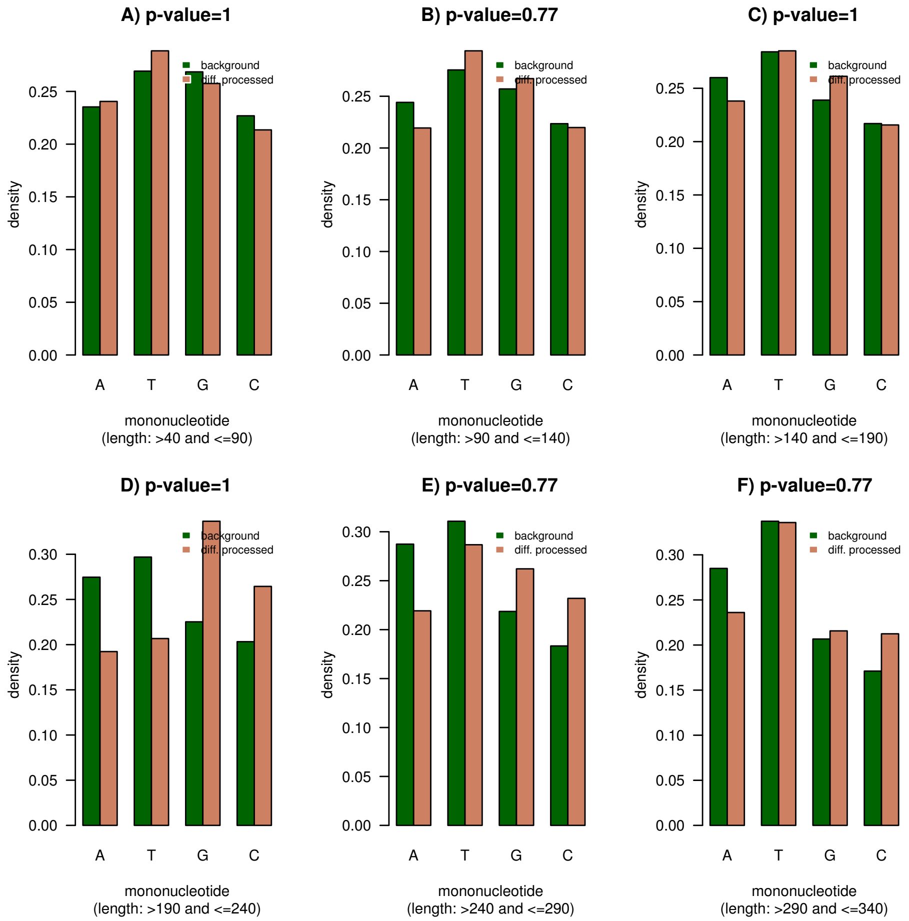

Supplementary Figure S8: Density distribution of four mononucleotides between the loci where expression is observed in atleast one of the N cell lines (background) and differentially processed loci. The two density distributions were compared individually for each of the six bins of block group length (A-F) using Kolmogorov-Smirnov test, p-value < 0.01. We observed no significant difference in the mononucleotide content between the differentially processed and background loci suggesting that differential processing identified at a locus is not effected by the technical artifacts imposed during the sequencing process by the mononucleotide content of a transcript.

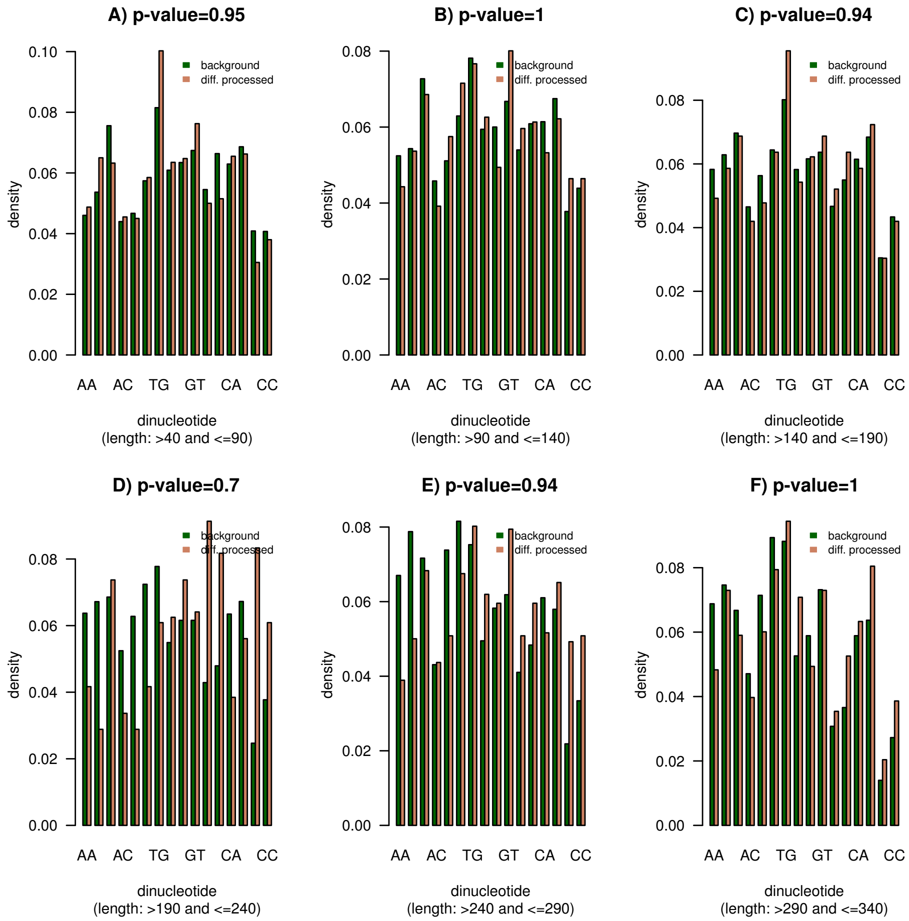

Supplementary Figure S9: Density distribution of sixteen dinucleotides between the loci where expression is observed in atleast one of the N cell lines (background) and differentially processed loci. The two density distributions were compared individually for each of the six bins of block group length (A-F) using Kolmogorov-Smirnov test, p-value < 0.01. We observed no significant difference in the dinucleotide content between the differentially processed and background loci suggesting that differential processing identified at a locus is not effected by the technical artifacts imposed during the sequencing process by the dinucleotide content of a transcript.

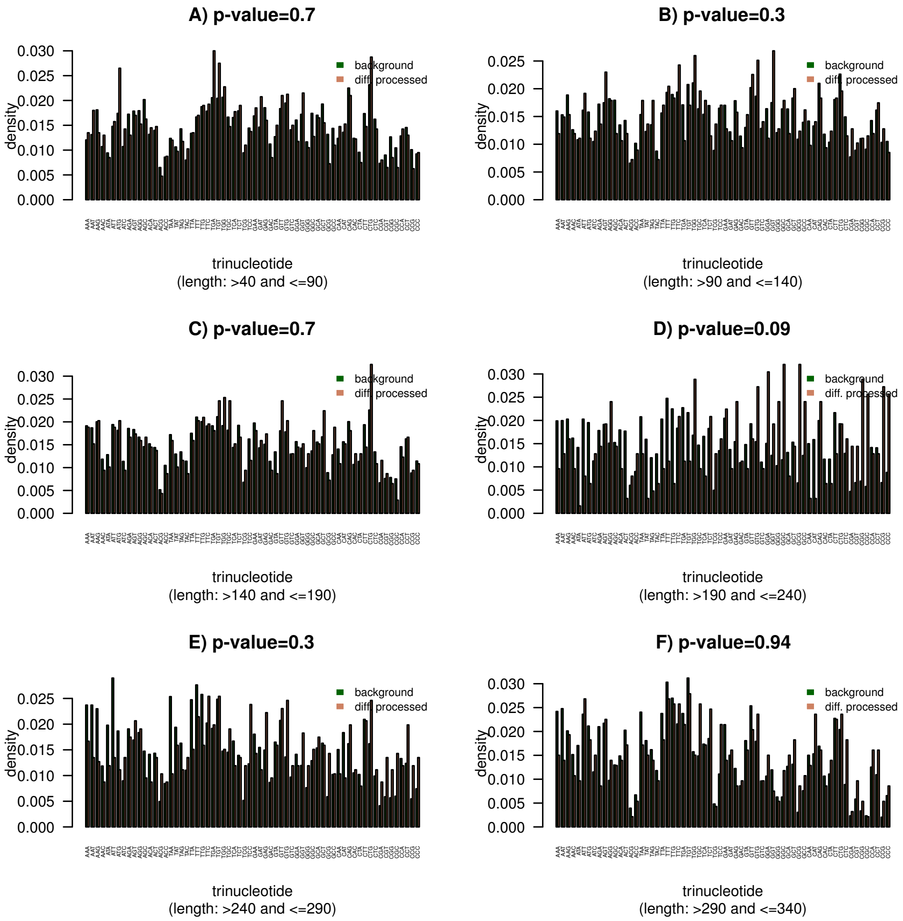

Supplementary Figure S10: Density distribution of sixty-four trinucleotides between the loci where expression is observed in atleast one of the N cell lines (background) and differentially processed loci. The two density distributions were compared individually for each of the six bins of block group length (A-F) using Kolmogorov-Smirnov test, *p*-value < 0.01. We observed no significant difference in the trinucleotide content between the differentially processed and background loci suggesting that differential processing identified at a locus is not effected by the technical artifacts imposed during the sequencing process by the trinucleotide content of a transcript.

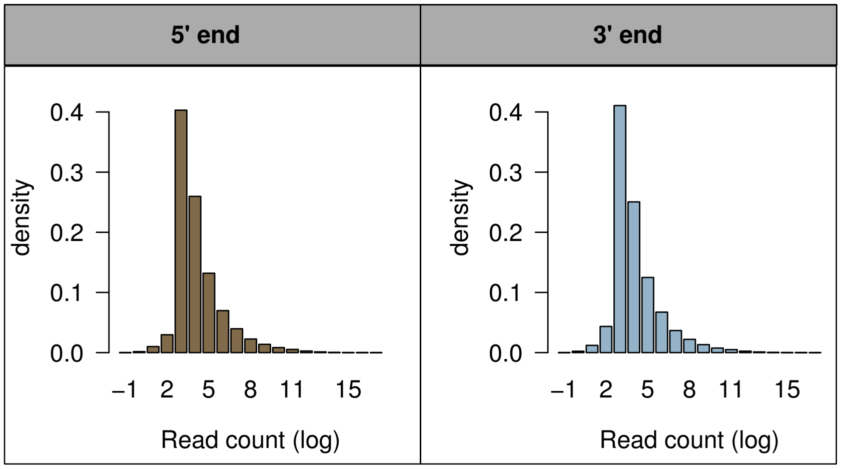

Supplementary Figure S11: Density distribution of read counts at the 5' and 3' end of the all block groups in ENCODE dataset. Each block group is divided into two halves, first starting from the 5' end to the mid point and second, from the mid point till the 3' end. Mean read count per nucleotide position is computed for each half of the block groups. We observed no significant difference in the density distribution of the mean read count (log) between the two ends of the block groups (Kolmogorov-Smirnov test, p-value$<$0.05). This suggests that there is no bias in the arrangement of reads relative to the 5' and 3' ends of the block groups.

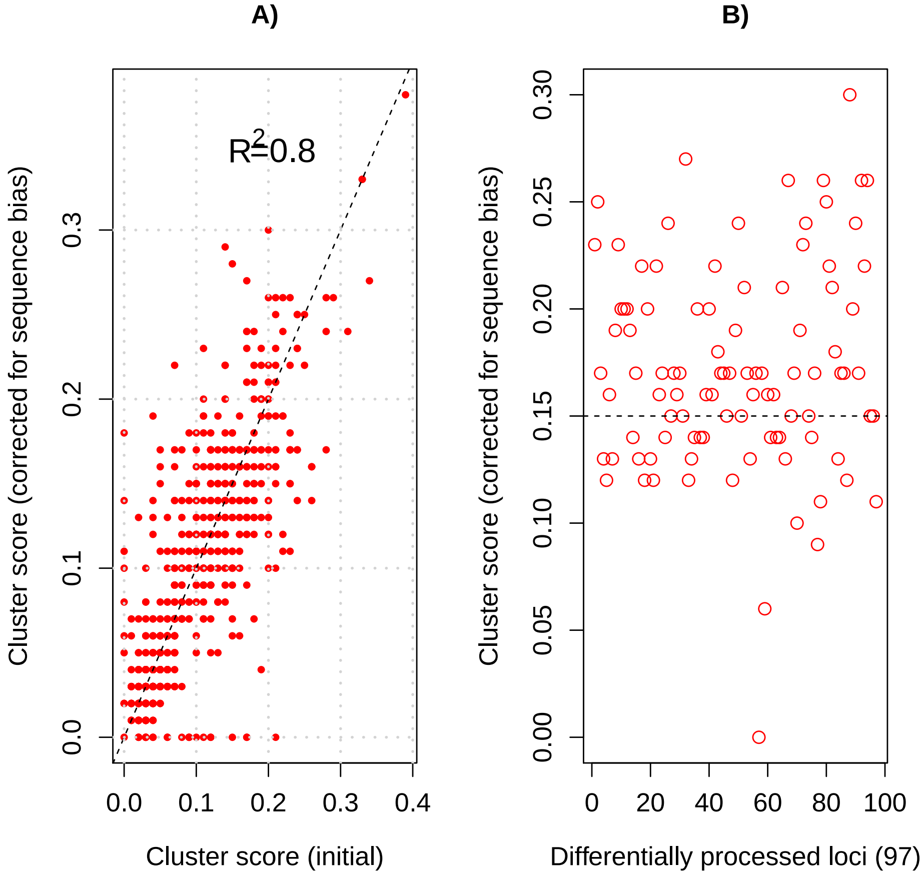

Supplementary Figure S12: Effect of the RNA-seq protocol bias per nucleotide position on the cluster scores. Any potential protocol bias is computed using seqbias, a R package that correct the frequency of mapped reads at each nucleotide position using a simple graphical model[13]. A) The cluster scores obtained after the analysis of 701 loci using our differential processing pipeline. The analysis is performed for read profiles at each of the 701 loci before and after correcting for sequence bias. We observed a high spearman's rank correlation of 0.8 suggesting that our pipeline is robust towards any potential sequence bias introduced during the RNA-seq experiments. b) Almost all of the 97 differentially processed loci identified upon the analysis of 701 loci showed a cluster score higher than or close to 0.15 (threshold for identifying differentially processed loci) after correcting for sequence bias.

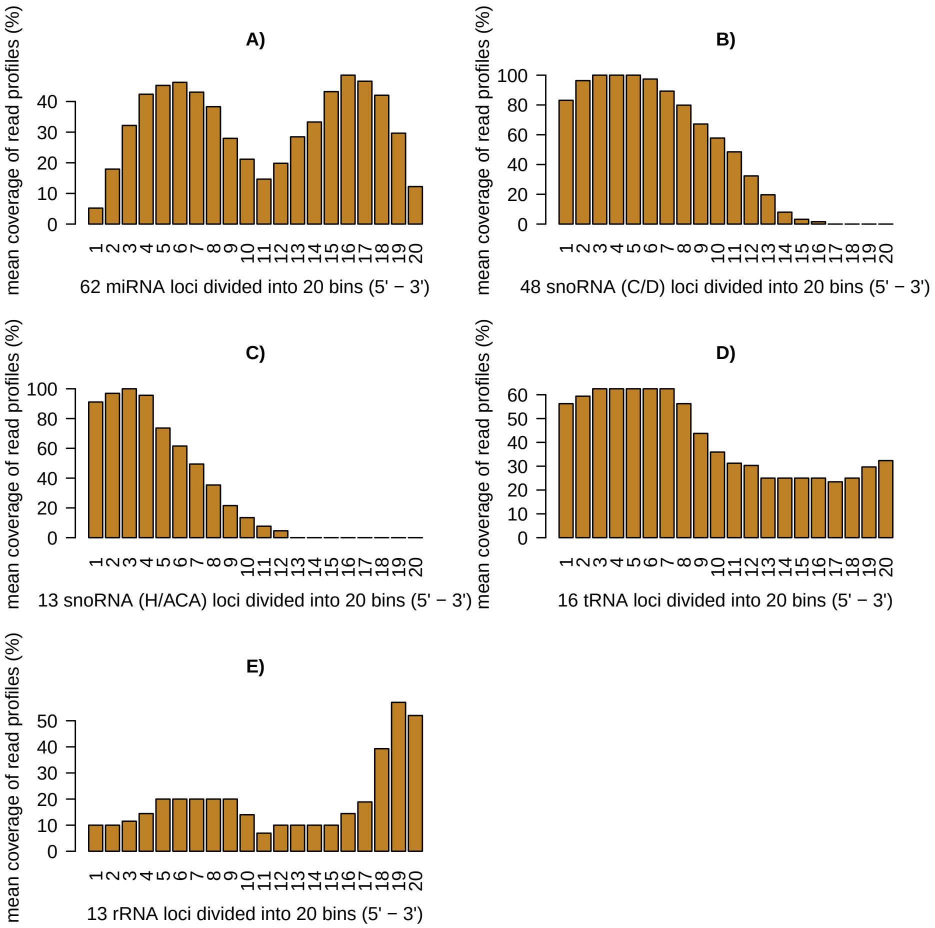

Supplementary Figure S13: The mean percentage coverage of read profiles at coherently processed loci annotated for non-coding RNAs (miRNA, snoRNA-C/D, snoRNA-H/ACA, tRNA and rRNA). The coverage for each class of ncRNA is computed by dividing the ncRNA gene coordinates into 20 equally sized bins followed by computing the percentage overlap of a read profile at each bin. The percentage overlap at each bin is then averaged over all the ncRNAs in each class. The read profile coverage is shown in 5'-3' direction. A) The read profile coverage for 62 miRNA genes. We observed no specific position bias in the arrangement of reads within the miRNA gene. B, C) The read profile coverage for the 61 snoRNA (48 C/D-box and 13 H/ACA-box) gene where most reads are precisely processed from the 5' end of snoRNA. D) The read profile coverage for the 16 tRNA genes where most reads are processed from the 5' end, albeit a smaller fraction of reads are also processed from the 3' end. E) The read profile coverage for 13 rRNA genes where most reads are processed from the 3' end of the gene. The position bias in the arrangement of reads from snoRNA, tRNA and rRNA is consistent with the findings of a recent study where these ncRNAs have been shown to asymmetrically produce small RNA fragments either from the 5' or 3' end[16].

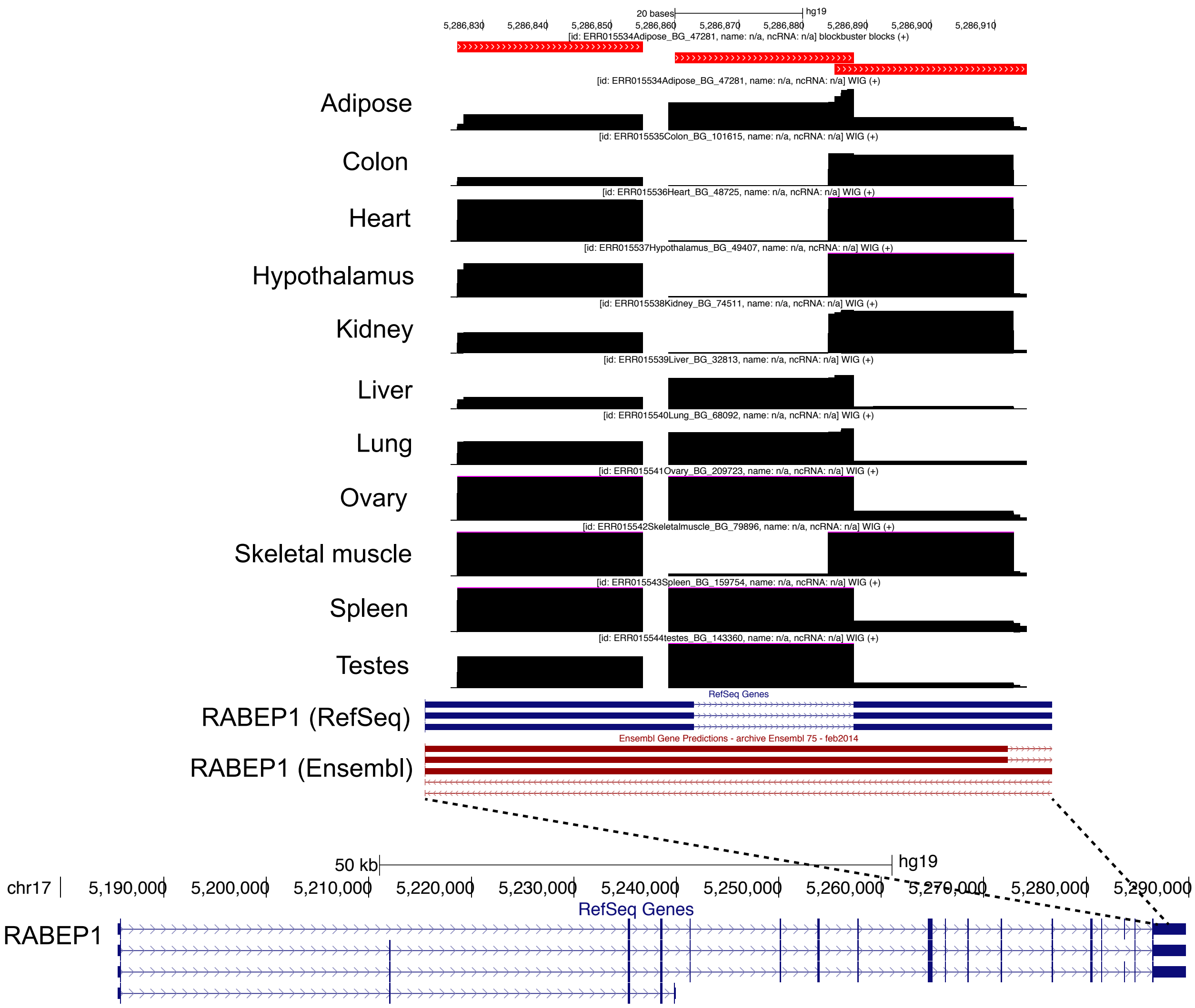

Supplementary Figure S14: An example of differential processing observed using total RNA-seq data[17]. We observed two distinct set of read profiles at the 3' UTR of *RABEP1* gene (bottom) across 11 human tissues (inset). While the expression corresponding to intron (blue thin line between two think lines) is predominant in six tissues (Adipose, Liver, Lung, Ovary, Spleen and Testes), it is negligible in rest of the five tissues (Colon, Heart, Hypothalamus, Kidney, Skeletal muscle). The height of read profiles have same scale in all 11 tissues. The example supports the expression of an alternate splice form of the transcript comprised of the intronic region in six tissues where it is expressed. Indeed, the latest Ensembl annotation (red) supports the existence of such an alternate form of the transcript. Also shown are all isoforms of *RABEP1* gene as per RefSeq annotation (below) along with a pointer to the position in 3' UTR (dotted lines) where the differential processing is observed.

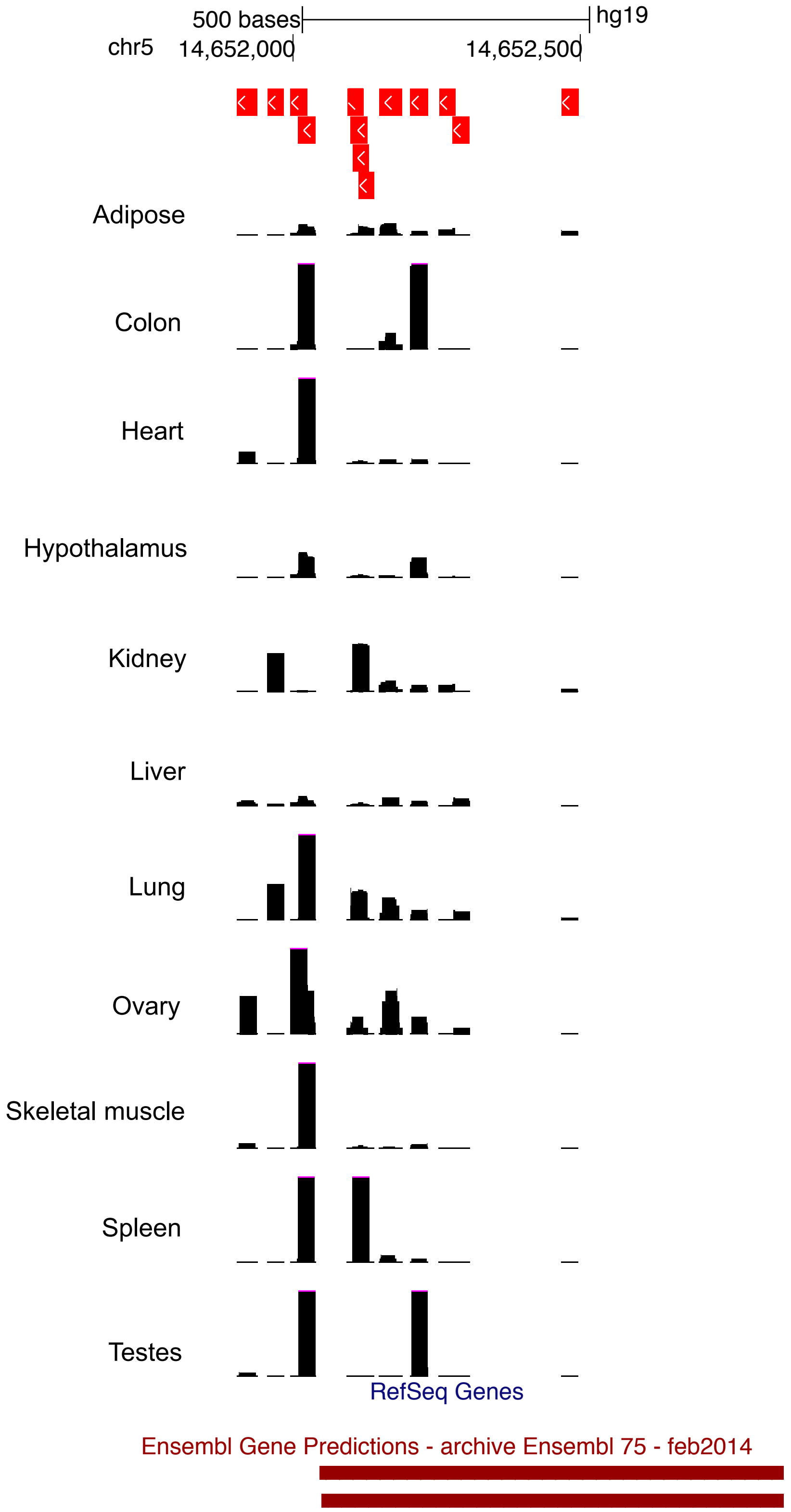

Supplementary Figure S15: An example of differential processing observed using total RNA-seq data[17]. We observed distinct set of read profiles at a genomic region partially overlapping with a pusedogene annotated by Ensembl. All the read profiles are at the same scale.

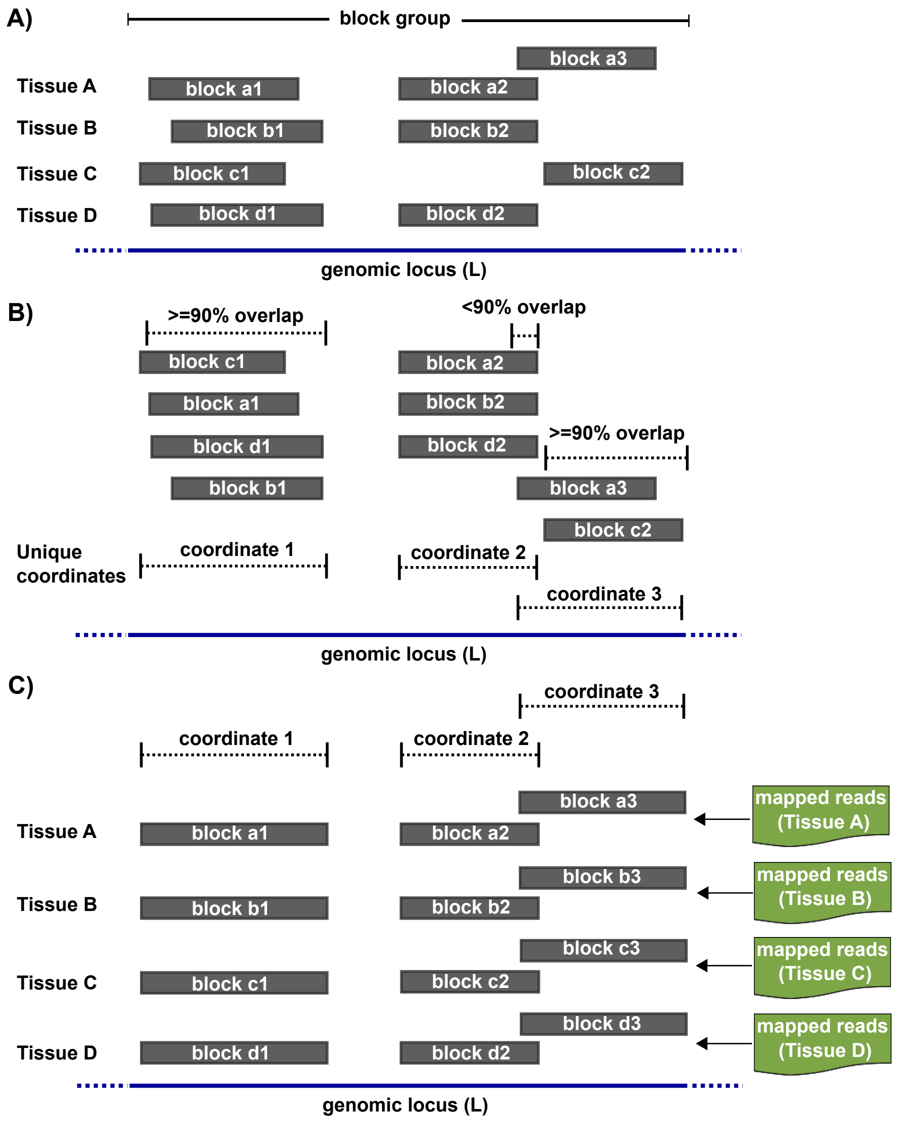

Supplementary Figure S16: Prefiltering steps in order to make read profiles comparable across different cell lines. A) For each genomic locus (L) where block groups are observed in all the cell lines under study, we determine if a block group is missing a read block. This can happen due to the fixed cut-off of 10% that we used to consider the expression of a block with respect to its parent block groups as significant (see methods in the manuscript). B) If missing, all the blocks at a locus (L) are arranged in the order of their start position and length such that blocks with lowest start position and length are placed first. A unique coordinate is initialized corresponding to the coordinate of first block group (lowest start position). Next, for each consecutive block starting from the lowest start position, the percentage overlap with the next closely spaced block is computed. If the overlap is $\geq 90\%$, the end position of the overlapping block is set as the end position of the unique coordinate, otherwise a new unique coordinate is initialized. C) For each of the unique coordinate identified, a dummy read with expression one is placed followed by retrieving all the reads that are mapped within this coordinate from the mapped read file.

Supplementary Tables

Supplementary Table S1: ENCODE dataset is comprised of 740 million reads, is part of the ENCODE project[18] and was prepared using short total-RNA (<200 nt) from nine different human cell lines reflecting tissues of diverse origin. Each cell line is having two biological replicates and prior to sequencing, these biological replicates have been grown and isolated independently. Dataset was downloaded in BAM format from ENCODE[18] and is composed of a uniform read length of 36 nt. A complete account of the sequencing and mapping procedure is described in[19]

**Source**[a]	**Replicate 1**				**Replicate 2**			
	**# reads**[b]	**# block groups**			**# reads**[b]	**# block groups**		
		all[c]	anno[d]	size factor[e]		all[c]	anno[d]	size factor[e]
Blood (GM12878)	49,280,641	6,295	1,005	1.43	56,439,584	7,422	1,068	1.73
Brain (SK-N-SH_RA)	48,773,897	6,008	1,087	1.54	48,394,385	5,252	1,059	1.18
Breast (MCF-7)	26,713,326	5,322	919	0.61	40,144,816	5,436	1,024	0.93
Cervix (HeLa-S3)	41,301,918	5,899	1,018	1.35	40,798,294	5,591	900	1.17
Epithelium (A549)	47,775,551	5,891	1,136	1.40	44,163,861	5,224	1,104	0.92
ES cell (H1-hESC)	35,965,377	4,953	1,018	0.65	33,651,242	5,494	994	0.57
Liver (HEPG2)	31,930,869	2,560	858	0.88	33,939,724	4,226	966	0.95
Lung (AG04450)	38,877,787	5,633	1,118	1.25	43,732,746	5,971	1,114	1.30
Skin (BJ)	37,649,014	2,883	1,000	0.57	41,022,882	4,412	969	0.69

[a]ENCODE cell lines are given within brackets. BAM files corresponding to both replicates of all the nine cell lines were downloaded from http://hgdownload.cse.ucsc.edu/goldenPath/hg19/encodeDCC/wgEncodeCshlShortRnaSeq/ (whole cell and TAP treated); GEO Id: GSE24565,
[b]number of mapped reads,
[c]the number of block groups defined by grouping closely spaced uniquely mapped reads as one block group or read profile (see methods in the manuscript and Supplementary Figure S1),
[d]block groups overlapping with annotated ncRNAs,
[e]size factor computed for each cell line to normalize the read counts with respect to the variable sequencing depth (Equation 3 in the manuscript).

Supplementary Table S2: Differential and coherently processed loci observed during analysis of 18 RNA-seq experiments from the ENCODE dataset.

**Annotation**	**DPL**[a]	**CPL**[b]	**Neither**[c]	**Total**
Unannotated	3	195	18	216
miRNA	22	62	35	119
snoRNA	31	61	84	176
tRNA	30	16	87	133
other ncRNA	11	19	27	57
Total	97	353	251	701

[a]Differentially processed loci, [b]Coherently processed loci, [c]Neither differential, nor coherently processed loci.

Supplementary Table S3: Nine miRNAs that are observed to exhibit arm switching (differentially processed). Five of these miRNA have also been shown to be arm switched in earlier studies (three in human and two in mouse).

miRNA	Coordinate	Cluster score[a]	Reference	Previously reported[b]
hsa-mir-33a	chr22:42296948-42297016	0.18	[20]	human (brain and liver)
hsa-mir-126	chr9:139565054-139565138	0.21	[20]	human (placenta and spleen)
hsa-mir-423	chr17:28444112-28444173	0.34	[21]	human (normal and tumor gastric tissue)
hsa-mir-30e	chr1:41220027-41220118	0.20	[22]	mouse (stomach and spleen)
hsa-mir-30a	chr6:72113254-72113324	0.21	[22]	mouse
hsa-mir-222	chrX:45606421-45606530	0.15		
hsa-mir-132	chr17:1953220-1953282	0.21		
hsa-mir-221	chrX:45605604-45605671	0.15		
hsa-mir-181b-2	chr9:127456002-127456062	0.24		

[a]Cluster score is a measure of differential processing at a locus that lies between 0 and 1,
[b]The organism and the tissues in which the arm switching has previously been reported.

Supplementary Table S4: Forty-six (thirty-nine unique) previously reported cases of the differential processing in miRNA (all arm switching). These cases were determined based on the analysis of short RNA-seq data from multiple human tissues or cell lines. Five of the miRNA fulfilled the criteria for differential processing analysis and four of them were also predicted in this study.

**miRNA[a]**	**Total**	**Reference[b]**	**miRNA fulfilling criteria[c]**	**Identified[d]**
mir-106b, hsa-mir-1307, hsa-mir-16, hsa-mir-1975[$], hsa-mir-223[#], hsa-mir-23b, hsa-mir-365	7	[23]	None	NA
hsa-mir-136, hsa-mir-361, hsa-mir-423, hsa-mir-376b, hsa-mir-1307	5	[21]	hsa-mir-423	Yes
hsa-mir-30c-2, hsa-mir-30b, hsa-mir-126, hsa-mir-106b, hsa-mir-377, hsa-mir-382, hsa-mir-511-1, hsa-mir-193b, hsa-mir-500a, hsa-mir-505, hsa-mir-548h-3, hsa-mir-664, hsa-mir-1307	13	[24]	hsa-mir-30b	Yes
hsa-mir-103-2, hsa-mir-126, hsa-mir-136, hsa-mir-154, hsa-mir-190, hsa-mir-1975, hsa-mir-19b-1, hsa-mir-29b-2, hsa-mir-3065, hsa-mir-337, hsa-mir-33a, hsa-mir-340, hsa-mir-361, hsa-mir-377, hsa-mir-411, hsa-mir-449b, hsa-mir-454, hsa-mir-483, hsa-mir-498, hsa-mir-519e, hsa-mir-545	21	[20]	hsa-mir-33a<br>hsa-mir-126<br>hsa-mir-361	Yes<br>Yes<br>No

[a]Previously reported cases of differential processing in miRNA; [#]Supported by wet-lab based experimental methods; [$]dead entry in miRBase,
[b] [23] studied arm-switching in ESC (Embyonic stem cells), Leukemic blood, Prostate and Color cell lines;
[21] studied arm-switching in normal and tumor gastric tissues;
[24] studied arm-switching between normal and tumor liver tissues;
[20] studied arm-switching between ten human tissues (Brain, Heart, Kidney, Liver, Lung, Ovary, Placenta, Spleen, Testes and Thymus).
[c]MiRNA fulfilling criteria such that they are expressed in both replicates of all nine cell lines analyzed in this study.
[d]If the miRNA is also identified as differentially processed in this study.

Supplementary Table S5: Read profiles are compared between two RNA-seq experiments performed on the same or different tissues in same and different laboratories, respectively. Most genomic loci ($\sim$95%) have similar read profiles between experiments performed on same tissue in same as well as different laboratory, as suggested by an alignment score of $\geq$0.6 and $\geq$0.55, respectively. As expected, a higher proportion of genomic loci are observed to have read profiles with low scores of $<$0.6 and $<$0.55 between experiments performed on different tissues in both same as well as different laboratory, respectively.

**Category**	**#All**[e]	**#Compared**[f]	**deepBlockAlign score $<$0.6**	***p*-value**[g]
Same tissues (same laboratory)[a]	1,458	838	41 (5%)	2.2e-23
Different tissues (same laboratory)[b]	1,513	697	172 (25%)	
**Category**	**#All**[e]	**#Compared**[f]	**deepBlockAlign score $<$0.55**	***p*-value**[g]
Same tissues (different laboratory)[c]	2,925	199	9 (5%)	2.1e-10
Different tissues (different laboratory)[d]	5,897	175	59 (34%)	

[a]Tissue 1: Blood (GSM605625, biological replicate 1); Tissue 2: Blood (GSM605625, biological replicate 2),
[b]Tissue 1: Blood (GSM605625); Tissue 2: Brain (GSM897070),
[c]Tissue 1: Testes (GSM475281); Tissue 2: Testes (GSM539877),
[d]Tissue 1: Testes (GSM475281); Tissue 2: Germinal center B-cells (GSM539846),
[e]Total number of genomic loci where the read profiles are observed in either of the two tissues,
[f]Total number of genomic loci where the read profiles are compared between the two tissues,
[g]Computed using Fisher's exact test as *fisher.test(matrix(c(41,838,172,697), nrow=2), alternative="l")$p.value* and *fisher.test(matrix(c(9,199,59,175), nrow=2), alternative="l")$p.value*).

Supplementary Table S6: Performance measure of differential processing analysis pipeline. It is evaluated by comparing the cluster scores from loci analyzed by using both the biological replicates from ENCODE dataset together (combined) and individually (separate).

**Combined**[a]	**Rep1**[b]	**Rep2**[c]	**TP**[d]	**TN**[e]	**FP**[f]	**FN**[g]	**Sensitivity**	**Specificity**	**MCC**[h]
97	116	-	69	557	47	28	0.71	0.92	0.59
97	-	128	69	545	59	28	0.71	0.90	0.55

[a]Differentially processed loci identified on combined analysis,
[b]Differentially processed loci identified on independent analysis of replicate 1,
[c]Differentially processed loci identified on independent analysis of replicate 2,
[d]Differentially processed loci identified on both combined and independent analysis,
[e]Non-differentially processed loci identified on both combined and independent analysis.,
[f]Differentially processed loci identified on independent analysis only,
[g]Differentially processed loci identified on combined analysis only,
[h]Matthew's Correlation Coefficient

Supplementary Table S7: Distinct frequency of the 'short and precise' block groups between 353 Coherently Processed Loci (CPL) and rest of the 348 loci that are analyzed for differential processing using the ENCODE dataset, respectively. Most block groups from CPL were observed to be short and precisely processed (Fisher's exact test).

**Category**	**# block groups[a]**		**Total[b]**	***p*-value[c]**
	short and precise	others		
CPL (353)	5,371 (85%)	983 (15%)	6,354 (353 x 18)	5.8e-243
Others (348)	1,875 (30%)	4,389 (70%)	6,264 (348 x 18)	

[a]short and precise block groups are composed of one read block, have low entropy ($\leq$2) and are $\leq$40 nt in length,
[b]total number of block groups or read profiles corresponding to 353 coherently processed and 348 remaining loci, respectively. For each locus, we have 18 block groups corresponding to the nine cell lines (two replicates each),
[c]Fisher's exact test.

Supplementary Table S8: Raz dataset (GEO id: GSE25028) is comprised of 6 RNA-Seq experiments performed on ribosome free total-RNA extracted from human liver cell line, replicated over six times, by independent scientists. Dataset was downloaded from EBI ArrayExpress database[25] and is composed of variable read length from 25 to 58 nt long.

SRA Id	Description	# reads[a]	# block groups		size factor[c]
			all[b]	anno[c]	
SRR133598	replicate 1, scientist 1	16,266,881 (77)	8,731	660	1.01
SRR133572	replicate 2, scientist 1	15,531,679 (75)	7,605	622	0.89
SRR133622	replicate 1, scientist 1	18,972,463 (69)	10,676	700	1.09
SRR133605	replicate 2, scientist 1	18,628,572 (53)	21,109	601	0.71
SRR133586	replicate 1, scientist 2	24,424,488 (64)	14,326	914	1.28
SRR133595	replicate 2, scientist 2	20,925,798 (69)	12,579	895	1.16

[a]reads obtained after quality filter. Percentage of mapped reads are given in brackets,
[b]the number of block groups defined by grouping closely spaced uniquely mapped reads as one block group or read profile (see methods in the manuscript and Supplementary Figure S1),
[c]block groups overlapping with annotated ncRNAs,
[d]size factor computed for each cell line to normalize the read counts with respect to the variable sequencing depth (Equation 3 in the manuscript).

References

1. Torarinsson, E. *et al.* Comparative genomics beyond sequence-based alignments: RNA structures in the ENCODE regions. *Genome research* **18**, 242–251 (2008).

2. Karolchik, D. *et al.* The UCSC table browser data retrieval tool. *Nucleic Acids Res.* **32**, D493–D496 (2004).

3. Hoffman, M. M. *et al.* Integrative annotation of chromatin elements from ENCODE data. *Nucleic acids research* **41**, 827–841 (2013).

4. Ernst, J. & Kellis, M. Discovery and characterization of chromatin states for systematic annotation of the human genome. *Nature biotechnology* **28**, 817–825 (2010).

5. Hoffman, M. M. *et al.* Unsupervised pattern discovery in human chromatin structure through genomic segmentation. *nature methods* **9**, 473–476 (2012).

6. Barrett, T. *et al.* NCBI GEO: archive for functional genomics data sets - 10 years on. *Nucleic acids research* **39**, D1005 (2011).

7. Langenberger, D. *et al.* deepblockalign: a tool for aligning rna-seq profiles of read block patterns. *Bioinformatics* **28**, 17–24 (2012).

8. Li, J., Jiang, H. & Wong, W. Method modeling non-uniformity in short-read rates in RNA-Seq data. *Genome Biol.* **11**, R25 (2010).

9. Zheng, W., Chung, L. & Zhao, H. Bias detection and correction in RNA-Sequencing data. *BMC bioinformatics* **12**, 290 (2011).

10. Hansen, K., Brenner, S. & Dudoit, S. Biases in illumina transcriptome sequencing caused by random hexamer priming. *Nucleic acids research* **38**, e131 (2010).

11. Benjamini, Y. & Speed, T. Summarizing and correcting the GC content bias in high-throughput sequencing. *Nucleic Acids Research* (2012).

12. Dohm, J., Lottaz, C., Borodina, T. & Himmelbauer, H. Substantial biases in ultra-short read data sets from high-throughput dna sequencing. *Nucleic acids research* **36**, e105–e105 (2008).

13. Jones, D. C., Ruzzo, W. L., Peng, X. & Katze, M. G. A new approach to bias correction in rna-seq. *Bioinformatics* **28**, 921–928 (2012).

14. Anders, S. & Huber, W. Differential expression analysis for sequence count data. *Genome Biol* **11**, R106 (2010).

15. Langenberger, D. *et al.* Evidence for human microRNA-offset RNAs in small RNA sequencing data. *Bioinformatics* **25**, 2298–2301 (2009).

16. Li, Z. *et al.* Extensive terminal and asymmetric processing of small RNAs from rRNAs, snoRNAs, snRNAs, and tRNAs. *Nucleic Acids Research* **40**, 6787–6799 (2012).

17. Castle, J. C. *et al.* Digital genome-wide ncRNA expression, including SnoRNAs, across 11 human tissues using polyA-neutral amplification. *PloS one* **5**, e11779 (2010).

18. Consortium, E. P. *et al.* A users guide to the encyclopedia of dna elements (encode). *PLoS Biol* **9**, e1001046 (2011).

19. Fejes-Toth, K. *et al.* Post-transcriptional processing generates a diversity of 5-modified long and short rnas. *Nature* **457**, 1028–1032 (2009).

20. Cloonan, N. *et al.* MicroRNAs and their isomiRs function cooperatively to target common biological pathways. *Genome Biol* **12**, R126 (2011).

21. Li, S.-C. *et al.* miRNA arm selection and isomir distribution in gastric cancer. *BMC genomics* **13**, S13 (2012).

22. Ro, S., Park, C., Young, D., Sanders, K. & Yan, W. Tissue-dependent paired expression of miRNAs. *Nucleic acids research* **35**, 5944–5953 (2007).

23. Kuchenbauer, F. *et al.* Comprehensive analysis of mammalian mirna* species and their role in myeloid cells. *Blood* **118**, 3350–3358 (2011).

24. Li, S.-C. *et al.* MicroRNA 3'end nucleotide modification patterns and arm selection preference in liver tissues. *BMC systems biology* **6**, S14 (2012).

25. Parkinson, H. *et al.* Arrayexpress updatean archive of microarray and high-throughput sequencing-based functional genomics experiments. *Nucleic acids research* **39**, D1002–D1004 (2011).

Graphical plots of 97 differentially processed loci (DPL)

Please click here to download graphical plots of 97 DPL as a single pdf file.

Graphical plots of 158 annotated coherently processed loci (CPL)

Please click here to download graphical plots of 158 annotated CPL as a single pdf file.

Graphical plots of 195 unannotated coherently processed loci (CPL)

Please click here to download graphical plots of 195 unannotated CPL as a single pdf file.
